# AlleleProfileR: A versatile tool to identify and profile sequence variants in edited genomes

**DOI:** 10.1371/journal.pone.0226694

**Published:** 2019-12-26

**Authors:** Arne A. N. Bruyneel, Alexandre R. Colas, Ioannis Karakikes, Mark Mercola

**Affiliations:** 1 Stanford Cardiovascular Institute, Stanford School of Medicine, Stanford, CA United States of America; 2 Department of Medicine, Division of Cardiovascular Medicine, Stanford School of Medicine, Stanford, CA, United States of America; 3 Sanford Burnham Prebys Medical Discovery Institute, La Jolla, CA, United States of America; 4 Department of Cardiothoracic Surgery, Stanford School of Medicine, Stanford, CA, United States of America; Tulane University Health Sciences Center, UNITED STATES

## Abstract

Gene editing strategies, such as zinc-finger nucleases (ZFNs), transcription activator-like effector nucleases (TALENs), and clustered regularly interspaced short palindromic repeat/Cas9 (CRISPR/Cas9), are revolutionizing biology. However, quantitative and sensitive detection of targeted mutations are required to evaluate and quantify the genome editing outcomes. Here we present AlleleProfileR, a new analysis tool, written in a combination of R and C++, with the ability to batch process the sequence analysis of large and complex genome editing experiments, including the recently developed base editing technologies.

## Introduction

**Fig 1:**
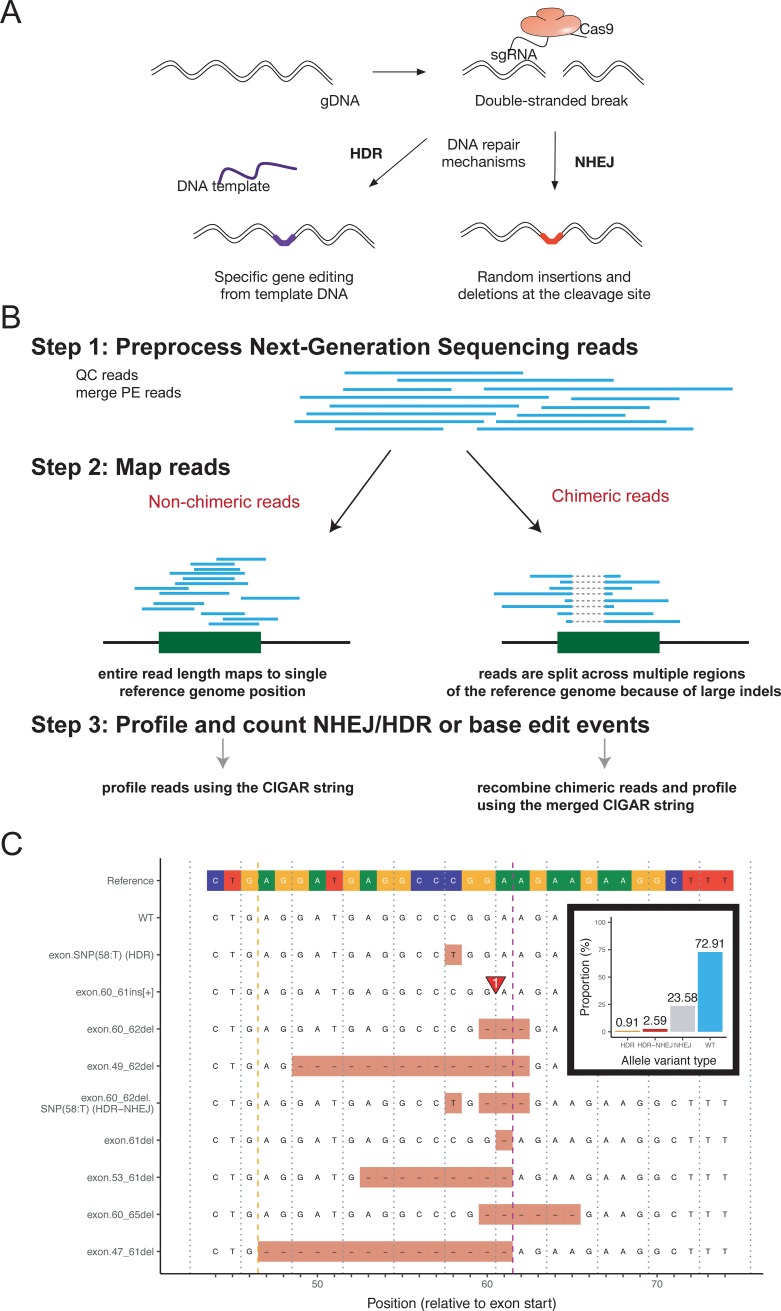
Overview of the AlleleProfileR algorithm and analysis output. (a) Overview of CRISPR technology and genome edit outcomes. (b) Analysis algorithm. NGS reads are preprocessed (QC, merging paired end reads), next the reads are mapped to the reference genome, and then the AlleleProfileR will profile the reads by analyzing the CIGAR strings. (c) Introduction of a TNNT2 mutation in human induced pluripotent stem cells: alignment of the top 10 identified alleles. The visualized region is Homo sapiens chromosome 1, GRCh38 bases 201363363 to 201363333, analyzable 67110 reads were processed spanning this region. The purple mark indicates the expected cut site, the yellow mark the analysis window boundaries, and the grey dashed lines the codons. (insert in b) Distribution of allele types, discriminating WT, NHEJ, HDR, and combinations of NHEJ-HDR for the introduction of the TNNT2 mutation.

Gene editing technologies are revolutionizing the way genetic experiments are conducted. It is now feasible to generate genome edited cell lines or animals in record time. Multiple designer nucleases have been developed for gene editing, including customized zinc-finger nucleases (ZFNs) [1], transcription activator-like effector nucleases (TALENs) [2] and clustered regularly interspaced short palindromic repeat/Cas9 (CRISPR/Cas9) [3]. These nucleases generate double-stranded breaks (DSBs) at particular sites in the genome that are subsequently repaired by non-homologous end joining (NHEJ) or homology-directed repair (HDR) [4, 5]. NHEJ creates random insertions/deletions or point mutations at the point of DSB that often result in frame shifts or loss-off-function of the gene. In contrast, HDR can create precise genetic alterations based on compatible DNA templates, such as the sister chromatids, homologous chromosomes, or exogenous DNA (Fig 1A). As a result, both the NHEJ and HDR pathways have been exploited extensively in research to generate transgenic animals and cell lines.

Genome editing using the HDR pathway enables the precise insertion of new or the repair of existing mutations. For example, induced pluripotent stem cells (iPSCs) paired with genome editing strategies are revolutionizing how inherited diseases are studied, including cardiac diseases [6], by enabling the generation of isogenic control lines. Moreover, base editing technologies that irreversibly convert one base into another without DSB, have recently been reported [7, 8]. In contrast, the insertions and deletions (indels) created by NHEJ have been widely used to generate KO *in vitro* and *in vivo* models across various species [9–14]. Importantly, it has become feasible to easily generate triple or quadruple KO animal models by multiplexed gene targeting [3, 9]. Multiple genes with redundant functions can be targeted directly by utilizing pooled single-guide RNAs (sgRNAs), and thus generate triple or even quadruple knockouts in a short period of time using *in vivo* NHEJ. Indeed, we recently generated a quadruple knock-out mouse embryo lacking all four *Id* (*Inhibitor of DNA binding*) genes (*Id1-4*) in the mouse by injecting eight sgRNAs and Cas9 mRNA into fertilized mouse oocytes [15]. Two guides per gene were used to increase the likelihood of creating null-alleles, given the likelihood of in-frame indels when only using one guide [16].

The potential applications of gene editing are numerous but are hindered by the limitations of existing quantitative technologies to quantify NHEJ/HDR or base editing efficiency and profile the generated mutants. Multiple tools have been developed in the recent years to analyze NHEJ/HDR outcomes and base editing experiments, including Cas-Analyzer [17], CRISPR-GA [18], CRISPResso [19], AGEseq [20], CrispRVariants [21], EditR [22], and BE-Analyzer [23]. However, we found that the current analysis tools could not accommodate our needs as they could not analyze chimeric reads caused by large deletions where the resulting reads align to two regions in the genome (up and downstream of the deletion). Hence all data in the quadruple *Id* gene KO study were analyzed manually [15]. Anticipating that such complex gene targeting experiments will become more common, we designed and implemented a versatile genome editing analysis pipeline using an R-package that can analyze the various types of experiments, termed AlleleProfileR.

## Methods

### Availability of software

AlleleProfileR is provided as open-source software and can be installed locally from source (GitHub, https://github.com/abruyneel/AlleleProfileR). Alternatively, AlleleProfileR can be utilized using the Docker container platform (Docker Hub, https://hub.docker.com/r/abruyneel/alleleprofiler). The Docker image provides a standalone environment to run AlleleProfileR which includes several external bioinformatics tools, this avoids dependency issues and helps users to get going immediately. A step-by-step guide on how to conduct analyses can be found in S1 File.

### Input data supplied by the user and preprocessing of sequencing data

AlleleProfileR requires four types of data to process a gene editing experiment: a reference genome, next generation sequencing (NGS) reads, gene-of-interest loci and configurations. The reference genome needs to be supplied in fasta format, but it is not necessary to provide the entire genome or chromosome, a large enough region spanning the sequenced region is sufficient. As only BAM files are accepted as input sequencing files, raw reads need to be preprocessed first which involves preprocessing, merging, and aligning the reads to the reference. Users can execute these preprocessing steps using their own favorite tools or processing pipeline, alternatively AlleleProfileR can apply these instructions to batches of files directly by linking to external software, such as fastp [24] for FASTQ preprocessing, PEAR [25], FLASH [26], or fastq-join for paired-end read merging [27], and BWA [28] for read alignment, making the analysis of large experiments easier. For example, following code snippet will perform QC using fastp, merge paired-end reads using PEAR, map the reads using BWA and save the resulting BAM files in their respective input folders.

# load the location of the fastq files in the input folders

# directory structure should be provided as files/input/sample.name/.

samples <- AlleleProfileR.read.folders(type = "fastq")

# preprocess the files, this step will perform quality control using fastp, merge # paired-end reads using PEAR, and map the reads using BWA.

AlleleProfileR.preprocess(samples, index = "files/index/frag.fa",

                                                                             method.qc = "fastp", params.qc = "",

                                                                             method.merge = "pear", params.merge = "-v 30",

                                                                             method.map = "bwa", subset = NULL)

Finally, AlleleProfileR requires configuration specifics with respect to the genomic regions of interest (for example: start and stop locations, targeted cut site) as well as specifics as to how AlleleProfileR should process the results. These configuration settings can be supplied using the AlleleProfileR.setup command, which accepts many parameters. Essential configuration settings are *samples*, *genes*, and *index*. The samples parameter supplies the samples for analysis, the gene table contains the coordinates of the genes of interest, and index is the reference genome. Optional parameters include among others *cutoff*, the minimum percentage occurrence required for reporting, and *ignore*.*snp* and *ignore*.*single*, Booleans indicating whether SNPs or alleles with only a single occurrence should be ignored, respectively.

# load the location of the bam files in the input folders

samplestable <- AlleleProfileR.read.folders(type = "bam")

# set configuration

crispr_config <- AlleleProfileR.setup(samples = samplestable,

                                                                             genes = "files/config/example_genes.csv",

                                                                             index = "files/index/frag.fa",

                                                                             cutoff = 0,

                                                                             ignore.snp = F,

                                                                             ignore.single = F)

### Processing reads and variant calling

AlleleProfileR will analyze all reads in the BAM file that span the region of interest, determine their allele name and count their occurrences. AlleleProfileR performs variant calling based on the alignment annotations, i.e. by assessing the CIGAR string of the BWA alignment. This string records the extent to which the read matches with the reference: (mis)matched bases are indicated as ‘M’, whereas deleted or inserted bases are represented as ‘D’ or ‘I’, respectively, which allows to pinpoint and extract the gene-editing events. However, chimeric reads or split reads, which occur when a sequencing read aligns to two distinct portions of the genome and are indicative of structural variation, require a more complex analysis procedure, and may arise when DSB-mediated NHEJ produces large indels with the same reads being mapped both downstream and upstream of the event site. AlleleProfileR deals with these reads by reassembling the individual reads, based on the truncation annotations performed by the aligner. The aligner will designate non-aligned parts of chimeric reads as clipped; soft (S) or hard (H), depending on whether these bases were removed from the sequence in the alignment record. In one pair of the chimeric read alignments, part of the sequence will be mapped, part will be clipped, in the other alignment of the pair, the clipped section will be mapped, absent any further indels, enabling the deconvolution of the chimera inducing event. The tutorial in S1 File incorporates *in silico* experiments demonstrating the performance of the algorithm, such as deletions ranging from 5 to 120 bases resulting in chimeric reads (Fig D in S1 File) and more complex chimeric reads with insertions within large deletions, such as the experiment in Fig E in S1 File where up to 120 scrambled bases were inserted at the deletion site of 120 bases.

### Impact annotation and output provided by the software

Based on the gene region information in the configuration, and on the variant calling output, AlleleProfileR will also assess the impact of the variant by assessing various metrics, such as start codon presence, frameshifts, indels within the coding sequence, premature stop codons, etc. Following code snippet will process all data using the aforementioned configuration:

# process files and determine allelic variants

AlleleProfileR.batch(crispr_config, cores = 3, subset = NULL)

All analyses, both at the level of individual reads as well as identified alleles is written to csv files in the output folder. AlleleProfileR can generate various summary statistics to summarize the experimental output, such as the incidence of WT, NHEJ, HDR reads. HDR is quantified as proportion of reads that perfectly match the HDR template. If other variants in addition to the HDR variant are present in a given read, it will be flagged as a combination of HDR and NHEJ (HDR-NHEJ). All non-HDR or HDR-NHEJ reads different from WT are considered NHEJ. Base edit efficiency is quantified at the level of base conversion of individual bases rather than alleles, as neighboring off-target bases may also be edited to a certain extend. AlleleProfileR incorporates various plotting options such as alignments for NHEJ/HDR experiments and sequence logo plots for base editing experiments [29] to aid in the interpretation and visualization of the data.

## Results and discussion

### Design of the software

AlleleProfileR is a tool to determine gene edit (NHEJ or HDR) or base edit efficiency from NGS data. The software development hypothesis was to resolve the issue of chimeric reads resulting from large deletions when using multiple guides targeting a small region in the genome and enabling batch operation whilst simultaneously making the tool broadly applicable and flexible. The algorithm was implemented in a combination of R and C++ using Rcpp [30] and is available as R-package for local installation or as a stand-alone Docker image. The analysis takes place in three main steps (Fig 1B). First, NGS reads are preprocessed, merged, and aligned. Secondly, the reads are processed using the annotations provided by the aligner, to determine variant name and impact. Based on the known start and stop location of the exon, AlleleProfileR will determine to what extent the coding sequence was affected by the indels or SNPs. For example, frameshift or mutation-induced stop codons and cryptic coding frames are identified. Moreover, AlleleProfileR determines the incidence of NHEJ/HDR or base edit efficiency. Finally, the results are summarized and can be presented using a variety of plotting tools (Fig 1C). Overall, the power of AlleleProfileR is its batch operation capabilities, compatibility with chimeric reads, and intuitive plots. Many current approaches, such as Cas-Analyzer [17], CRISPR-GA [18], CRISPResso [19], and AGEseq [20], rely on aggregated variant summaries which may be suitable to estimate cutting efficiency of guides, but is of little value to conduct genotype-phenotype assessments in edited cell lines or embryos where summary statistics are insufficient because of the incidence of in-frame indels or truncated proteins.

### Genome editing and HDR

Genome editing strategies can be used to introduce new genetic variants or reverse gene variants to their WT state. AlleleProfileR offers batch processing and summary statistics, in contrast to most current approaches that are limited to one sample and one genomic location [17–20]. To showcase the utility of AlleleProfileR we determined the HDR frequency of introducing a *TNNT2* mutation in human induced pluripotent stem cells (Fig 1C). Cas-Analyzer [17], which reports HDR in combination with NHEJ also as HDR, determined that the HDR efficacy was 2.8% for this experiment. However, only HDR-only events may be relevant when estimating the HDR efficacy of a protocol. Hence, AlleleProfileR reports HDR+NHEJ and HDR-only events separately: 0.9% and 2.6% for accurate HDR and a mix of HDR and NHEJ, respectively.

### Large deletions and chimeric reads

**Fig 2:**
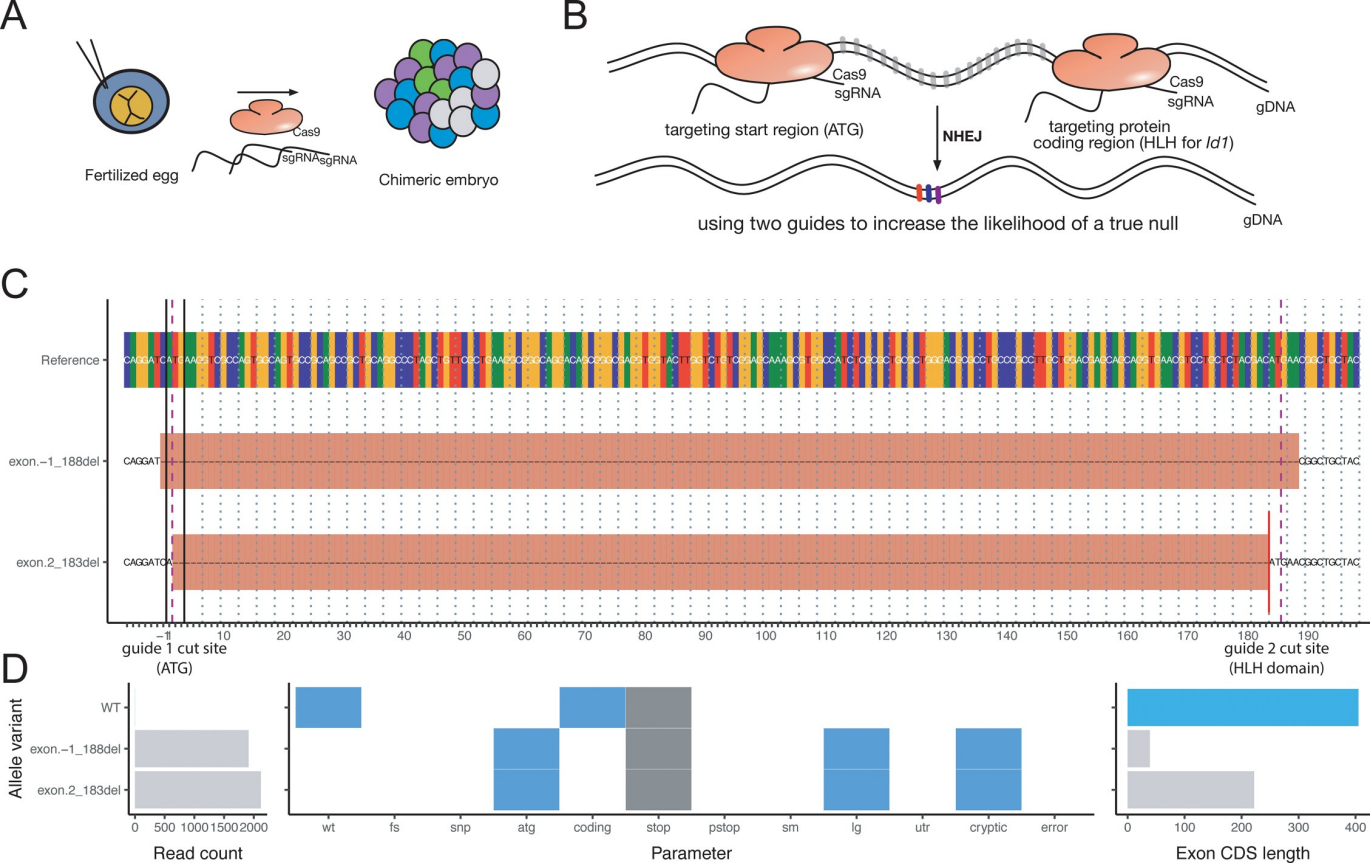
Overview of the in vivo experiments of Cunningham et al. [15] that were reanalyzed using AlleleProfileR. (a) Schematic of the experimental setup: fertilized eggs were treated with Cas9 and two guide RNAs per targeted protein. The inefficient nature of CRISPR gives rise to chimeric embryos comprised of cells with varying degrees of editing. (b) Two guides per target gene were designed to target the start codon (ATG) and the protein coding region (HLH domain) in order to increase the likelihood of creating a null allele. The likely gene edits were expected to be indels at either or both sites, or deletion of the entire region between both guides. (c) Gene edit outcome of one of the embryos (targeting of *Id1*, GRCm38 mm10, chromosome 2, bases 152736334 to 152736538 were visualized). The purple mark indicates the expected cut sites for both guides used (separated by 185 bases). Large deletions were observed spanning the region of the two guides. (d) (left) Two alleles with high abundance were detected, but no WT allele. (middle) Characterization of the alleles. The x-axis represents different metrics that were scored, a white box indicates ‘false’, whereas a blue box indicates ‘true’, gray boxes indicate not applicable. WT indicates whether the coding sequence is WT, FS whether frame-shifts are present, SNP whether SNPs are present, ATG whether the start codon is destroyed, CODING whether the allele is coding for a protein using the normal start codon, STOP whether the stop codon was destroyed, PSTOP whether a premature stop codon is present, SM whether small indels are present, LG whether large indels are present, UTR whether indels are present in the UTR regions, CRYPTIC whether cryptic coding sequences are present, and ERROR whether the algorithm failed to make a determination. Please refer to the tutorial for a more detailed description and worked-out examples illustrating these metrics. (right) The length of the coding sequence. Both alleles of this embryo have a large deletion spanning the region targeted by the two guides, including the start codon, and both alleles have smaller coding region present in the residual sequence of this exon. In particular, the 2–183 del variant gives rise to a cryptic start codon one bp downstream of the original start codon. If expressed, it would give rise to a truncated Id1 variant with intact HLH domain.

We reanalyzed some of the data from the Cunningham *et al*. [15] study. In contrast to established tools such as CrispRVariants [21], AlleleProfileR can characterize chimeric deletions (Figs F and G in S1 File). CRISPR-DAV can detect chimeric alleles, but is limited to one guide region [31]. Two guides, here targeting either the ATG or the start of the HLH domain, are often used to increase the likelihood of a null allele. Nonetheless, in-frame indels or truncated proteins that retain some function can arise from two-guide mediated NHEJ. Therefore, AlleleProfileR was designed to estimate the likely consequence of an indel, by determining whether the start or stop codons are affected, or frame shift was induced, or putative cryptic coding sequences are present. AlleleProfileR analysis of the Cuningham et al. [15] dataset revealed that the 2–183 deletion gave rise to a cryptic start codon one bp downstream of the original start codon, and hence an Id1 variant with truncated N-terminal end but intact HLH domain (Fig 2C and 2D). Such variants might have had residual function and could therefore explain the partial phenotypes that were seen in some of the embryos [15].

### Base editing

**Fig 3:**
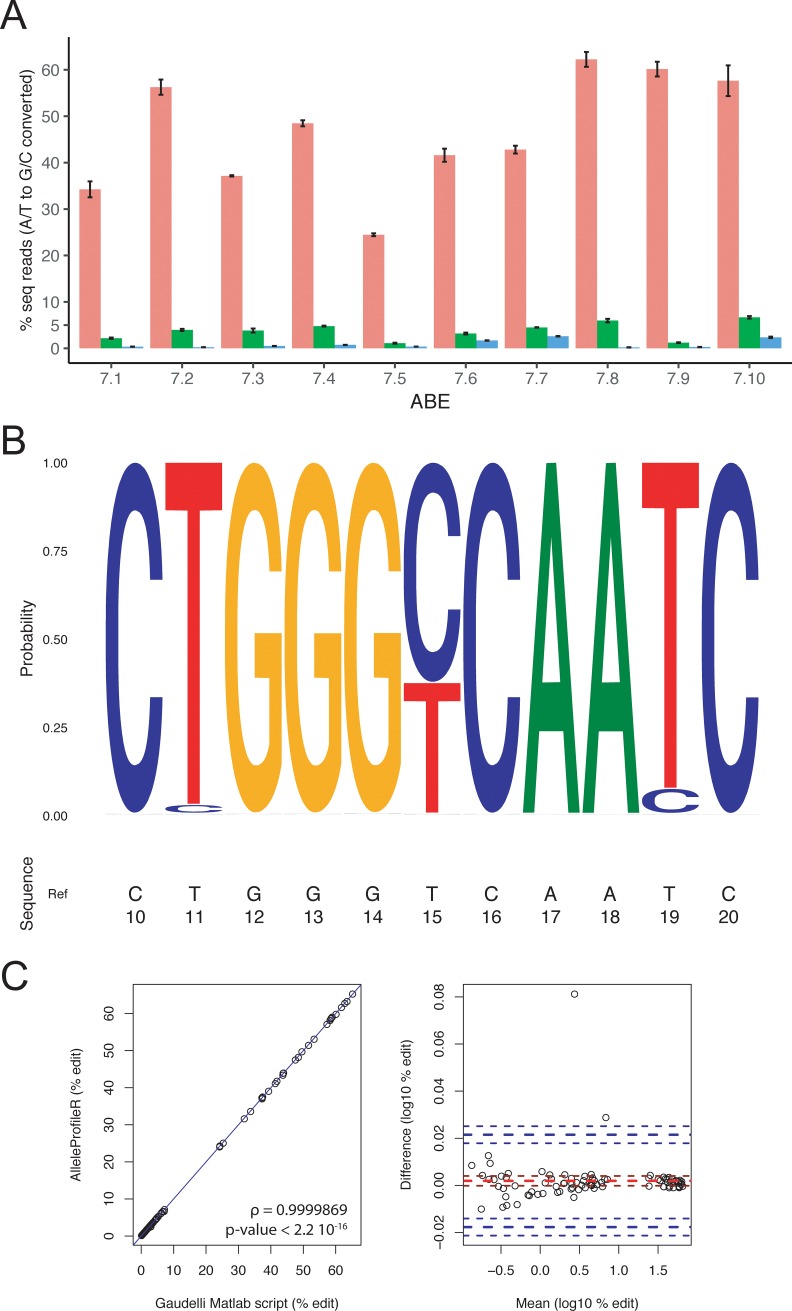
Analysis of adenine base editors (ABE) outcomes. (a) On-target base editing (pink), off-target edits at two sites (green: off-target 1, blue: off-target 2) for site 6 as described in [7] (profiled region: Homo sapiens chromosome 9, GRCh38, bases 107422299 to 107422348). The bar charts represent mean ± standard error (three replicates) for different ABE variants (7.1 to 7.10). (b) Sequence logo plot of sample site 6_ABE7.10_HEK293T_rep 3 (plotted region: GRCh38, chromosome 9, 107422323 to 107422333). Position 15 is the on-target edit, position 19 and 11 are off-target positions 1 and 2, respectively. (c) Comparison between the base-edit efficacies as determined by AlleleProfileR and the Gaudelli et al. [7] matlab script. All deposited fastq files for site 6 [7] were analyzed using both methods for both on-target as well as off-target base-edit events. (left) correlation plot of the result obtained by using the two methods, the blue line represents the identify line. (right) Bland-Altman plot relating the difference between the two methods to the average result of the two methods on the log10 transformed data. The mean difference and its 95% confidence limits are indicated in red, whereas the ± two standard deviation markers and their confidence limits are labeled blue. An example and step-by-step guide to reproduce this figure can be found in the supplementary tutorial, Figures M to O in S1 FIle.

Furthermore, we reanalyzed some of the data from the study of Gaudelli *et al*. [7] describing the adenine base editors (ABEs) that mediate the conversion of A•T to G•C in genomic DNA. The NGS data from one genomic site and different ABE were downloaded from the NCBI Sequence Read Archive database and were reanalyzed using AlleleProfileR (Fig 3A). AlleleProfileR’s plotting tools provide a convenient graphical representation of both on-target as well as off-target base editing events (Fig 3B). Our analysis agreed with the reported analysis using a custom local alignment script implemented in Matlab [7] (Fig 3C). Briefly, the results of AlleleProfileR correlate perfectly with the analysis using the original script (ρ = 0.9999869, p-value < 2.2e-16) and the difference between both methods in this dataset is small. These small differences can likely be explained by the different ways the methods deal with sequencing noise. Alternate tools have also been reported such as BE-Analyzer [23] which provides a convenient online tool to determine base edit efficacy. However, the tool can only analyze one sample at the time and has limited downstream analytical capabilities as it lacks batch operation capabilities.

## Conclusion

In summary, AlleleProfileR offers a robust workflow for processing *in vitro* or *in vivo* genome editing experiments. The advantages of AlleleProfileR over currently established tools is that it operates in batch mode and efficiently deals with chimeric reads caused by using two guides and provides multiple visualization tools for displaying the results, in contrast to most current tools that allow the analysis of single samples, small indels, or single guides.

## Supporting information

S1 File Tutorial on how to install and use AlleleProfileR. The tutorial additionally incorporates validation experiments and comparisons with other tools.
(PDF)

## References

[1] Kim YG, Cha J, Chandrasegaran S. Hybrid restriction enzymes: zinc finger fusions to Fok I cleavage domain. Proc Natl Acad Sci U S A. 1996;93(3):1156–60. 10.1073/pnas.93.3.1156 8577732 PMC40048

[2] Boch J, Scholze H, Schornack S, Landgraf A, Hahn S, Kay S, et al Breaking the code of DNA binding specificity of TAL-type III effectors. Science. 2009;326(5959):1509–12. 10.1126/science.1178811 . 19933107

[3] Jinek M, Chylinski K, Fonfara I, Hauer M, Doudna JA, Charpentier E. A programmable dual-RNA-guided DNA endonuclease in adaptive bacterial immunity. Science. 2012;337(6096):816–21. Epub 2012/06/28. 10.1126/science.1225829 . 22745249 PMC6286148

[4] Jasin M, Rothstein R. Repair of strand breaks by homologous recombination. Cold Spring Harb Perspect Biol. 2013;5(11):a012740 Epub 2013/11/01. 10.1101/cshperspect.a012740 24097900 PMC3809576

[5] Chiruvella KK, Liang Z, Wilson TE. Repair of double-strand breaks by end joining. Cold Spring Harb Perspect Biol. 2013;5(5):a012757 Epub 2013/05/01. 10.1101/cshperspect.a012757 23637284 PMC3632057

[6] Kaur B, Perea-Gil I, Karakikes I. Recent Progress in Genome Editing Approaches for Inherited Cardiovascular Diseases. Curr Cardiol Rep. 2018;20(7):58 Epub 2018/06/02. 10.1007/s11886-018-0998-3 . 29860642

[7] Gaudelli NM, Komor AC, Rees HA, Packer MS, Badran AH, Bryson DI, et al Programmable base editing of A•T to G•C in genomic DNA without DNA cleavage. Nature. 2017;551(7681):464–71. Epub 2017/10/25. 10.1038/nature24644 29160308 PMC5726555

[8] Komor AC, Kim YB, Packer MS, Zuris JA, Liu DR. Programmable editing of a target base in genomic DNA without double-stranded DNA cleavage. Nature. 2016;533(7603):420–4. Epub 2016/04/20. 10.1038/nature17946 27096365 PMC4873371

[9] Wang H, Yang H, Shivalila CS, Dawlaty MM, Cheng AW, Zhang F, et al One-step generation of mice carrying mutations in multiple genes by CRISPR/Cas-mediated genome engineering. Cell. 2013;153(4):910–8. Epub 2013/05/02. 10.1016/j.cell.2013.04.025 23643243 PMC3969854

[10] Crispo M, Mulet AP, Tesson L, Barrera N, Cuadro F, dos Santos-Neto PC, et al Efficient Generation of Myostatin Knock-Out Sheep Using CRISPR/Cas9 Technology and Microinjection into Zygotes. PLoS One. 2015;10(8):e0136690 Epub 2015/08/25. 10.1371/journal.pone.0136690 26305800 PMC4549068

[11] Sato M, Miyoshi K, Nagao Y, Nishi Y, Ohtsuka M, Nakamura S, et al The combinational use of CRISPR/Cas9-based gene editing and targeted toxin technology enables efficient biallelic knockout of the α-1,3-galactosyltransferase gene in porcine embryonic fibroblasts. Xenotransplantation. 2014;21(3):291–300. Epub 2014/02/21. 10.1111/xen.12089 . 24919525

[12] Edvardsen RB, Leininger S, Kleppe L, Skaftnesmo KO, Wargelius A. Targeted mutagenesis in Atlantic salmon (Salmo salar L.) using the CRISPR/Cas9 system induces complete knockout individuals in the F0 generation. PLoS One. 2014;9(9):e108622 Epub 2014/09/25. 10.1371/journal.pone.0108622 25254960 PMC4177897

[13] Cho SW, Lee J, Carroll D, Kim JS. Heritable gene knockout in Caenorhabditis elegans by direct injection of Cas9-sgRNA ribonucleoproteins. Genetics. 2013;195(3):1177–80. Epub 2013/08/26. 10.1534/genetics.113.155853 23979576 PMC3813847

[14] Shalem O, Sanjana NE, Hartenian E, Shi X, Scott DA, Mikkelson T, et al Genome-scale CRISPR-Cas9 knockout screening in human cells. Science. 2014;343(6166):84–7. Epub 2013/12/12. 10.1126/science.1247005 24336571 PMC4089965

[15] Cunningham TJ, Yu MS, McKeithan WL, Spiering S, Carrette F, Huang CT, et al Id genes are essential for early heart formation. Genes Dev. 2017;31(13):1325–38. Epub 2017/08/09. 10.1101/gad.300400.117 28794185 PMC5580654

[16] Zhou J, Shen B, Zhang W, Wang J, Yang J, Chen L, et al One-step generation of different immunodeficient mice with multiple gene modifications by CRISPR/Cas9 mediated genome engineering. Int J Biochem Cell Biol. 2014;46:49–55. Epub 2013/11/20. 10.1016/j.biocel.2013.10.010 . 24269190

[17] Park J, Lim K, Kim JS, Bae S. Cas-analyzer: an online tool for assessing genome editing results using NGS data. Bioinformatics. 2017;33(2):286–8. Epub 2016/08/24. 10.1093/bioinformatics/btw561 27559154 PMC5254075

[18] Güell M, Yang L, Church GM. Genome editing assessment using CRISPR Genome Analyzer (CRISPR-GA). Bioinformatics. 2014;30(20):2968–70. Epub 2014/07/01. 10.1093/bioinformatics/btu427 24990609 PMC4184265

[19] Pinello L, Canver MC, Hoban MD, Orkin SH, Kohn DB, Bauer DE, et al Analyzing CRISPR genome-editing experiments with CRISPResso. Nat Biotechnol. 2016;34(7):695–7. 10.1038/nbt.3583 27404874 PMC5242601

[20] Xue LJ, Tsai CJ. AGEseq: Analysis of Genome Editing by Sequencing. Mol Plant. 2015;8(9):1428–30. Epub 2015/06/06. 10.1016/j.molp.2015.06.001 . 26057235

[21] Lindsay H, Burger A, Biyong B, Felker A, Hess C, Zaugg J, et al CrispRVariants charts the mutation spectrum of genome engineering experiments. Nat Biotechnol. 2016;34(7):701–2. 10.1038/nbt.3628 . 27404876

[22] Kluesner MG, Nedveck DA, Lahr WS, Garbe JR, Abrahante JE, Webber BR, et al EditR: A Method to Quantify Base Editing from Sanger Sequencing. CRISPR J. 2018;1:239–50. 10.1089/crispr.2018.0014 . 31021262 PMC6694769

[23] Hwang GH, Park J, Lim K, Kim S, Yu J, Yu E, et al Web-based design and analysis tools for CRISPR base editing. BMC Bioinformatics. 2018;19(1):542 Epub 2018/12/27. 10.1186/s12859-018-2585-4 30587106 PMC6307267

[24] Chen S, Zhou Y, Chen Y, Gu J. fastp: an ultra-fast all-in-one FASTQ preprocessor. Bioinformatics. 2018;34(17):i884–i90. 10.1093/bioinformatics/bty560 30423086 PMC6129281

[25] Zhang J, Kobert K, Flouri T, Stamatakis A. PEAR: a fast and accurate Illumina Paired-End reAd mergeR. Bioinformatics. 2014;30(5):614–20. Epub 2013/10/18. 10.1093/bioinformatics/btt593 24142950 PMC3933873

[26] Magoč T, Salzberg SL. FLASH: fast length adjustment of short reads to improve genome assemblies. Bioinformatics. 2011;27(21):2957–63. Epub 2011/09/07. 10.1093/bioinformatics/btr507 21903629 PMC3198573

[27] Aronesty E. Comparison of Sequencing Utility Programs. The Open Bioinformatics Journal 2013;7:1–8. Epub 31/1/2013. 10.2174/1875036201307010001

[28] Li H, Durbin R. Fast and accurate long-read alignment with Burrows-Wheeler transform. Bioinformatics. 2010;26(5):589–95. Epub 2010/01/15. 10.1093/bioinformatics/btp698 20080505 PMC2828108

[29] Wagih O. ggseqlogo: a versatile R package for drawing sequence logos. Bioinformatics. 2017;33(22):3645–7. 10.1093/bioinformatics/btx469 . 29036507

[30] Eddelbuettel D, Francois R. Rcpp: Seamless R and C++ Integration. Journal of Statistical Software. 2011;40(8). 10.18637/jss.v040.i08

[31] Wang X, Tilford C, Neuhaus I, Mintier G, Guo Q, Feder JN, et al CRISPR-DAV: CRISPR NGS data analysis and visualization pipeline. Bioinformatics. 2017;33(23):3811–2. 10.1093/bioinformatics/btx518 . 28961906

