# Supplementary material for: AlleleProfileR: A versatile tool to identify and profile sequence variants in edited genomes
Source: PLoS One. 2019 Dec 26;14(12):e0226694. doi: 10.1371/journal.pone.0226694 (PMC6932767; doi:10.1371/journal.pone.0226694)
Supplement: S1 File — The tutorial additionally incorporates validation experiments and comparisons with other tools. (PDF) [file pone.0226694.s001.pdf]

AlleleProfileR Tutorial

A. A. N. Bruyneel et al.

Dec 2019

Introduction

This tutorial describes how to install, configure, and utilize the AlleleProfileR R-package for analyzing gene editing outcomes.

Getting started

The source of this package can be downloaded from github (https://github.com/abruyneel/AlleleProfileR). AlleleProfileR will be submitted to the CRAN soon. This package has several dependencies, including other R-packages (such as Bioconductor[1]) as well as external software. For analysing sequencing data, samtools[2], bwa[3], pear[4], and fastp[5], are needed (or similar tools). In addition, if you would like to conduct in silico experiments, wgsim[6] is also required.

```
# install R dependencies
install.packages(c("devtools","BiocManager"))
BiocManager::install(c("BiocGenerics", "Biostrings", "GenomicAlignments",
                       "GenomicRanges", "Rsamtools", "XVector"))

# install AlleleProfileR using devtools and github
devtools::install_github("abruyneel/AlleleProfileR")

# load AlleleProfileR
library(AlleleProfileR)
```

Alternatively, AlleleProfileR can be run from a docker container too, and can be downloaded from the Docker hub repository (abruyneel/alleleprofiler). The container is based on the rocker/verse image and deploys RStudio to use R, and also contains some of the external tools that can be used to process sequencing data, such as samtools (http://www.htslib.org) and bwa (http://bio-bwa.sourceforge.net/bwa.shtml). In addition, an example script and demo data is included.

To start the docker container:

```
docker run --rm -e PASSWORD=crispr -p 8787:8787 abruyneel/alleleprofiler
```

Open a web browser and browse to http://localhost:8787 or http://127.0.0.1:8787, logon on RStudio using the username 'rstudio' and password 'crispr', and run the example.R file.

A local folder (here ~\data) can be mounted to the Docker by adding the -v parameter to the command to initialize Docker. Use this strategy to run AlleleProfileR on your own datasets.

[1] https://bioconductor.org
[2] http://www.htslib.org
[3] http://bio-bwa.sourceforge.net/bwa.shtml
[4] http://www.exelixis-lab.org/web/software/pear
[5] https://github.com/OpenGene/fastp
[6] https://github.com/lh3/wgsim

Table A. Overview of the mutations inserted in the synthetic embryos.

Embryo	Gene	Description
1	NKX2.5	deleted CCCCAGCCC few bases near ATG
1	PLN	deleted T in ATG
1	TBX5	deleted 10bps
1	CAMK2D	delete T in ATG, insert G to make ATG in frame with end
2	NKX2.5	Mix of Embryo1 and WT
2	PLN	Mix of Embryo1 and WT
2	TBX5	Mix of Embryo1 and WT
2	CAMK2D	Mix of Embryo1 and WT
3	NKX2.5	WT
3	PLN	deleted TGCAAT...ATTTAT, inserted on deleted site: GGG
3	TBX5	inserted a T creating a stop
3	CAMK2D	WT
4	NKX2.5	Mix of Embryo1 and WT
4	PLN	Mix of deleted ACT post ATG, Embryo1 and WT
4	TBX5	Mix of 50 bp deletion (CAAGG...AGTGG) and deletion (CCC), Embryo 1 and WT
4	CAMK2D	Mix of Embryo1 and WT
5	NKX2.5	CCCCAG to CCCGAG
5	PLN	AAAAA to AATAA
5	TBX5	AATCAAG to AATGGAG
5	CAMK2D	ATGGCTTCG to ATGGCTTAG
Mix	NKX2.5	Mix of all
Mix	PLN	Mix of all
Mix	TBX5	Mix of all
Mix	CAMK2D	Mix of all
WT	NKX2.5	WT
WT	PLN	WT
WT	TBX5	WT
WT	CAMK2D	WT
dels	NKX2.5	series of dels
del120-ins	NKX2.5	series of ins in 120 bp del

```
docker run --rm -e PASSWORD=crispr -p 8787:8787
        -v ~/data:/home/rstudio/data abruyneel/alleleprofiler
```

Example data

To illustrate the use and potential application of AlleleProfileR, we will analyse a few example datasets. These synthetic samples were generated by inserting mutations into the genomic sequences of four genes relevant to cardiomyocytes (NKX2.5, TBX5, PLN, and CAMK2D, Table A) and simulating reads using wgsim. AlleleProfileR provides a wrapper function linking to wgsim to simulate paired-end reads: AlleleProfileR.simulate(). This function simulates paired-end reads from fasta files. If an array of filenames is given as filenames parameter, then the reads originating from all files in the array will be pooled.

```
AlleleProfileR.simulate(input.folder = "/.../files/input/wt", input.file = "wt.fa")
```

Table B. Configuration table of the genes of interest

Gene	Chr	Start	Stop	ATG	StopCodon	StartType	StartShift	StopType	StopShift	PCRStart	PCRStop	CutSites
NKX2.5	chr14	5415	5620	5424	5752	N	0	I	0	5000	6000	5600
PLN	chr10	6150	6350	6179	6335	N	0	N	0	5500	6700	NA
TBX5	chr5	600	750	633	723	I	0	I	2	400	1000	NA
CAMK2D	chr3	330	470	364	424	N	0	I	2	100	1000	NA

Configuration

File structure

AlleleProfileR requires a particular file structure for operation. In the working directory, a folder 'files' with 'config', 'index', 'input', 'output' as subfolders is required. The config folder must contain a csv file with details on the genes of interest (Table B). Reads are selected from the .bam-file for analysis if they span the region of interest as defined by the 'Start' and 'Stop' locations in the genes table. To assess whether the coding frame is altered, information on the theoretic start and stop codons of the exon need to be reported. 'StartType' or 'StopType' equals to N for normal codons: ATG, and TAG, TGA, and TAA respectively. 'PCRStart' and 'PCRStop' are the locations of the PCR primers used for amplification. 'CutSites' denotes the predicted cut positions of the editing strategy. If there are multiple cut sites, they should be separated by ';'.

The index folder must contain the reference genome in fasta format. It is not necessary to have the entire reference genome available. It is sufficient to provide the relevant chromosomes or the relevant regions within those chromosomes. The input folder must contain subfolders per sample comprising the fastq files or the bam files. The AlleleProfileR.read.folders() command scans the files/input folder for subfolders and .fastq.gz (type = "fastq") or .bam (type = "bam") files. The output folder will contain all the generated output, such as data tables and log files, but preprocessed fastq files and generated bam files will be saved into the sample input folders.

Configuration object

The analysis functions obtain their settings from a configuration list object, set using the AlleleProfileR.setup() command. By default, the sequence analysis scope of AlleProfileR is limited to the Start and Stop positions as defined in the gene configuration. The scope can be further limited by setting the cut.range value. This will restrict AlleleProfileR's analysis to this range around the cut site(s). Morover, a cutoff value (percent value, 0 to 1) can also be set for calling variants, such that infrequent variants or sequencing noise will be ignored. Variants occuring only once can be ignored by setting the ignore.single parameter to TRUE, SNPs will be ignored by setting ignore.snp to TRUE, and chimeric reads will be ignored by setting ignore.chimeric to TRUE. Finally, a list of alternate objects can be supplied to the alternate parameter to determine proportion homology-directed repair (HDR) in gene editing experiments.

```r
# read folders
samplestable <- AlleleProfileR.read.folders(type = "bam")

# set configuration
crispr_config <- AlleleProfileR.setup(samples = samplestable,
                                       genes = "files/config/example_genes.csv",
                                       index = "files/index/frag.fa",
                                       cutoff = 0,
                                       ignore.snp = F,
                                       ignore.single = T,
                                       ignore.chimeric = F,
                                       cut.range = 0,
                                       alternate = NULL,
                                       cutoff.large = 25,
```

```
                                     chimeric.max.insertion.size = 50,
                                     suppress.messages = F)
```

Basic analysis example

The basic analysis operation comprises following steps: (1) pre-process the reads, (2) align reads to reference, (3) determine allelic variants, and (4) plot the results and generate summary statistics. Step (1) and (2) can be completed using external software, or by using AlleleProfileR.

Process reads: merge and align to index

Typical read lengths are short (75-150 bp). However, when using two guides it is feasible to delete sequences larger than the read length. Additionally, the exon and genetic region of interest may be larger than the length of a typical read. If overlapping paired-end reads are merged first, the reads can be utilized for the detection of larger indels. In the current working example, we simulated paired-end fastq reads and utilized PEAR for merging the reads. Alternative algorithms may also be applied, but AlleleProfileR needs to be provided with single end or merged paired-end reads.

AlleleProfileR utilizes reference aligned reads generated by BWA, rather than local alignments. PEAR and BWA commands can be executed on the sample datasets directly from R, either on the entire files/input folder by setting the parameter subset to NULL, or on a selection of samples by setting subset to the indices of the samples (samplestable).

```
# this does every step at once
samples <- AlleleProfileR.read.folders(type = "fastq")
AlleleProfileR.preprocess(samples, index = "files/index/frag.fa",
                         method.qc = "fastp", params.qc = "",
                         method.merge = "pear", params.merge = "-v 30",
                         method.map = "bwa", subset = NULL)

# this is the step by step method

# do QC using fastp
samples <- AlleleProfileR.read.folders(type = "fastq")
AlleleProfileR.qc(samples, method = "fastp", params = "", subset = NULL)

# merge peaired-end reads using PEAR
samples <- AlleleProfileR.read.folders(type = "fastq-clean")
AlleleProfileR.merge(samples, method = "pear", params = "-v 40", subset = NULL)

# align reads to reference genome using BWA
samples <- AlleleProfileR.read.folders(type = "fastq-clean")
AlleleProfileR.map(samples, method = "bwa", index = "files/index/frag.fa", subset = NULL)

# set configuration
samplestable <- AlleleProfileR.read.folders(type = "bam")
crispr_config <- AlleleProfileR.setup(samples = samplestable,
                                     genes = "files/config/example_genes.csv",
                                     index = "files/index/frag.fa",
                                     cutoff = 0,
                                     ignore.snp = F,
                                     cut.range = 0,
                                     ignore.single = T,
                                     ignore.chimeric = F,
```

Table C. Overview of characterization parameters

Parameter	Description
WT	If TRUE, the CDS is WT
FS	If TRUE, a frame shift is present in the CDS
SNP	If TRUE, one or more SNPs were detected
ATG	If TRUE, the start codon was destroyed
CODING	If TRUE, the read is coding (start/stop detected)
STOP	If TRUE, the stop codon was destroyed
PSTOP	If TRUE, an in frame premature stop codon was detected
SM	If TRUE, a small indel is present in the CDS
LG	If TRUE, a large indel is present in the CDS
UTR	If TRUE, an indel is present in the UTR region
CRYPTIC	If TRUE, a cryptic variant is present
ERROR	If TRUE, the algorithm failed to assess this read

```
                                              cutoff.large = 25,
                                              chimeric.max.insertion.size = 250,
                                              suppress.messages = F)
```

Determine allelic variants

The AlleleProfileR.batch() function executes the variant determination algorithm on all data as set by the configuration or its parameters. The computation speed can be enhanced by increasing the cores value, which will split the task across multiple CPUs. The subset parameter allows the user to alter the queued files for analysis: all samples will be analysed if subset is set to NULL. Alternatively, to analyse a subset of samples for a selection of genes, subset should be set to a list containing a vector with the indices of the samples as first element, and a vector containing the indices of the genes of interest as second element.

```
# process files and determine allelic variants
AlleleProfileR.batch(crispr_config, cores=3, subset = NULL)
```

Summaries and plots

The variant calling algorithm writes all output to .csv-files which can be imported into R or any other software for further processing or plotting. AlleleProfileR contains several tools to visualize the results, as discussed in the sections below.

The AlleleProfileR.batch.summary() command plots an overview of the percentage WT sequence per embryo and per gene (default: param = “wt”), and lists the number of alleles detected (Figure A). Allele distributions, characterisation of the alleles and alignments for individual samples and genes can be plotted using AlleleProfileR.plot() command (Figure B). Allele characterization is reported using a boolean tile plot: blue indicates TRUE, white indicates FALSE, and gray indicates NA (Table C). Alignments can be plotted using AlleleProfileR.plot.alignment(), whereas AlleleProfileR.sample.distribution() and AlleleProfileR.sample.distribution.boolean() will plot only the distributions or characterization, respectively. Finally, the read depth for a certain characterization can be plotted using AlleleProfileR.plot.readdepth() (Figure C).

```
# general overview
AlleleProfileR.batch.recompute(crispr_config, cutoff = 0.005, ignore.single = T, top = 0)
AlleleProfileR.batch.summary(crispr_config, table=F, plot=T, subset = list(c(3:10),c(1:4)))
```

```
# plot alignments
AlleleProfileR.plot(crispr_config, 3, 1)
```

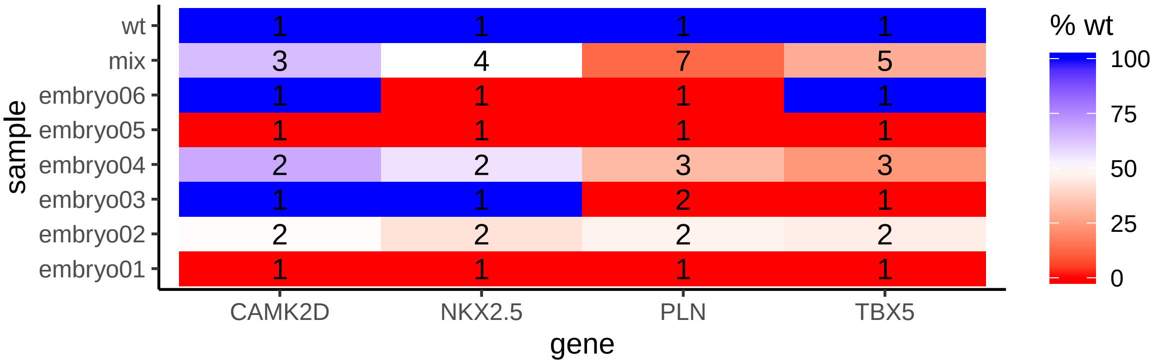

Fig A. Allelic variants overview plot generated by AlleleProfileR.batch.summary(). The color scale indicates the percentage WT per gene/sample combination. The label within each tile enumerates the number of alleles detected.

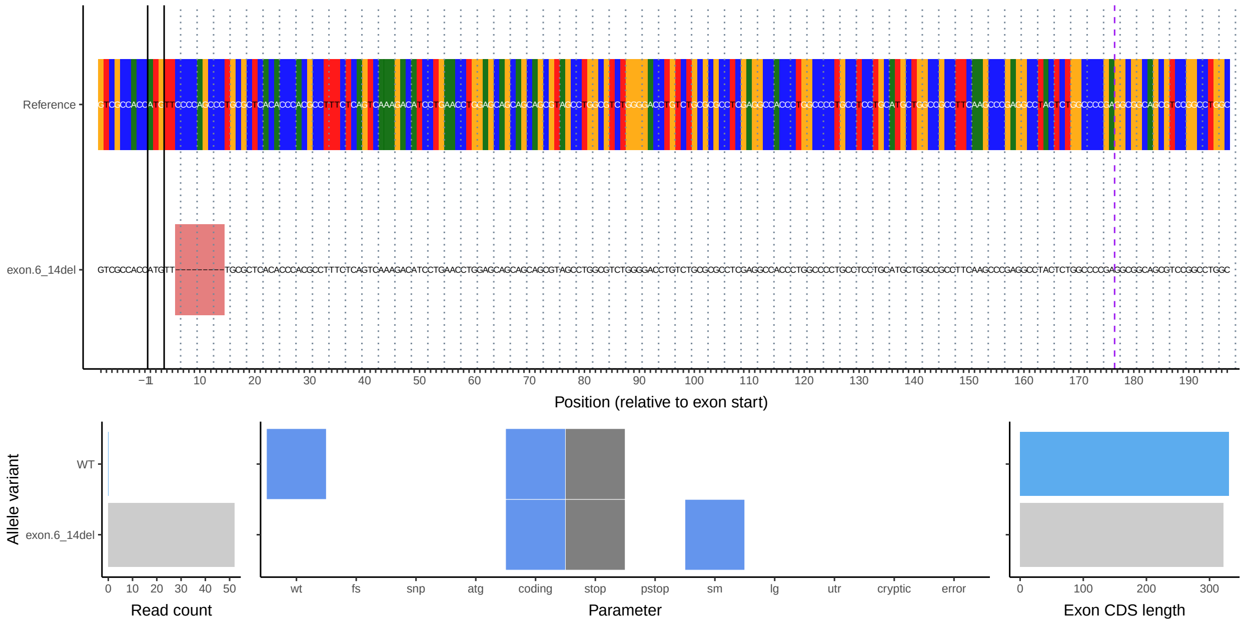

Fig B. Sequence alignment and characterization plot generated by AlleleProfileR.plot(). This sample has an in-frame deletion of 9 bps downstream of the start codon. Hence, expression of this variant allele will result in a truncated protein. As a result, AlleleProfileR determined that the allele is coding (CODING true) and has a small indel (SM true). STOP was set to NA as the stop codon falls outside of the region of interest and hence no determination was made.

```
# plot alignments
AlleleProfileR.plot.readdept(crispr_config, 3, 1)
```

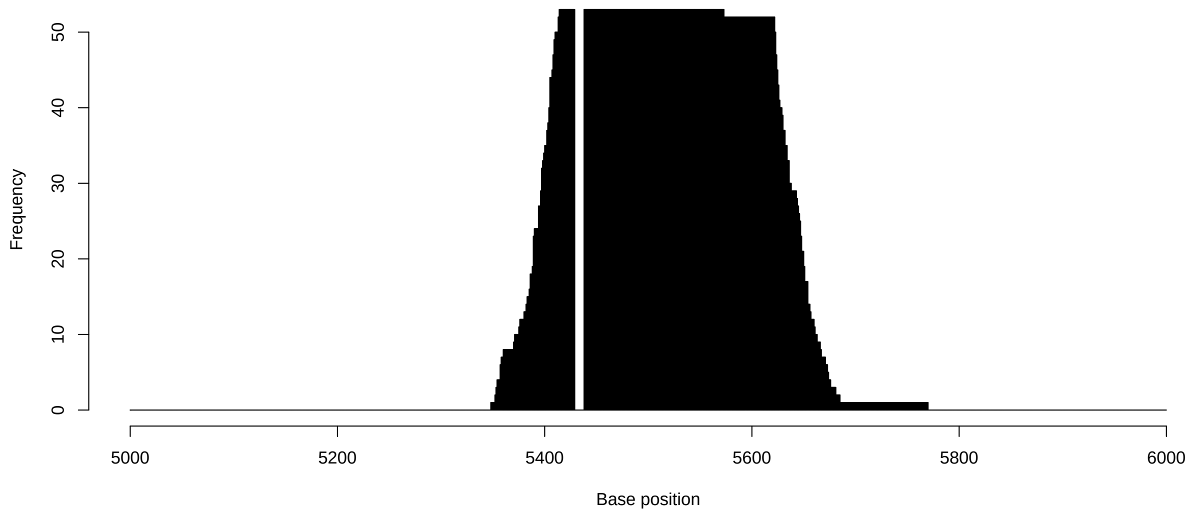

Fig C. Read depth plot of a sample with an in-frame deletion of 9 bps several bases downstream of the start codon.

Notes and applications

Gene editing technologies, such as clustered regularly interspaced short palindromic repeat/Cas9 (CRISPR/Cas9), offer several applications such as introducing random indels by non-homologous end-joining (NHEJ), or altering the genomic code using homology-directed repair (HDR) with a template. In addition, the genomic sequence can also be editing in a cut-independent fashion by base-editing enzymes[7].

NHEJ and chimeric reads

Several other tools have been developed to analyze NHEJ data, including CrispRVariants[8]. Here we compared the performance of CrisprVariants and AllelProfiler by analyzing the Synthetic data set 1 supplied by the CrispRVariants package. AlleleProfileR detects the same on-target alleles as CrispRVariants (Figure F), but additionally detects a chimeric variant which was excluded in CrispRVariants (Figure G).

To demonstrate the potential of AlleleProfileR in dealing with large indels, we generated some example datasets. For example, Fig. D represents the alignments of a series of deletions ranging from 5 to 120 bp. Whereas small deletions are captured by the aligner without inducing chimeras, the larger deletions, such as 100 bbp, induce chermic read alignments and are discarded by other tools. Similarly, Fig. E represents a series of insertions on the site of a 120 bp deletion, such large deletions and insertions similarly give rise to chimeric alignments.

[7] Gaudelli NM, Komor AC, Rees HA, Packer MS, Badran AH, Bryson DI, et al. Programmable base editing of A • T to G • C in genomic DNA without DNA cleavage. Nature. 551(7681), 464, 2017.

[8] Lindsay H, Burger A, Biyong B, Felker A, Hess C, Zaugg J, Chiavacci E, Anders C, Jinek M, Mosimann C, Robinson MD (2016). "CrispRVariants charts the mutation spectrum of genome engineering experiments." Nature Biotechnology, 34, 701–702. doi: 10.1038/nbt.3628.

```r
AlleleProfileR.plot(crispr_config, 2, 1, type = "count-only")
```

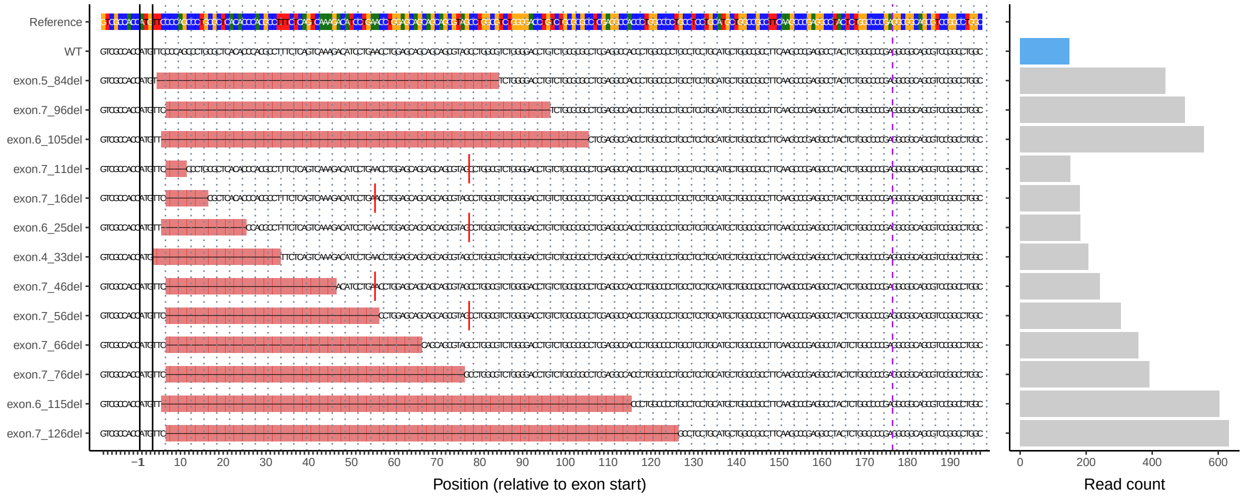

Fig D. Sequence alignment of a series of deletions ranging from 5 to 120 bases. If chimeric reads are excluded, deletions of 110 and 120 bp were not detected. The number of reads counted for all other variants were lower too.

```r
AlleleProfileR.plot(crispr_config, 1, 1, type = "count-only", max.insertion.size = 50)
```

Gene correction and alternative references

To quantify the degree of gene correction by HDR, the HDR template or alternative reference needs to be provided:

```r
# generate configuration information for an alternate reference
alternateinfo <- AlleleProfileR.alternatereference(crispr_config,
                     alternate = "files/index/alternate.fastq")
```

By adding the parameter 'alternate' to the plotting functions, the HDR allele(s) will be marked (Figure H).

```r
# plot alignments with alternate reference
AlleleProfileR.plot(crispr_config, 6, 2, alternate = alternateinfo, type = "count-only")
```

In addition, the proportions of NEHJ, HDR and mixtures of HDR and NHEJ can be computed and plotted (Figure I).

```r
# plot bar chart with mutation types
AlleleProfileR.plot.mutationtypes(crispr_config, 6, 2, alternate = alternateinfo, title = F)
```

```
## [[1]]
##       type     prop reads
## 1       WT 31.96721    39
## 2     NHEJ 36.88525    45
## 3      HDR 31.14754    38
## 4 HDR-NHEJ  0.00000     0
##
```

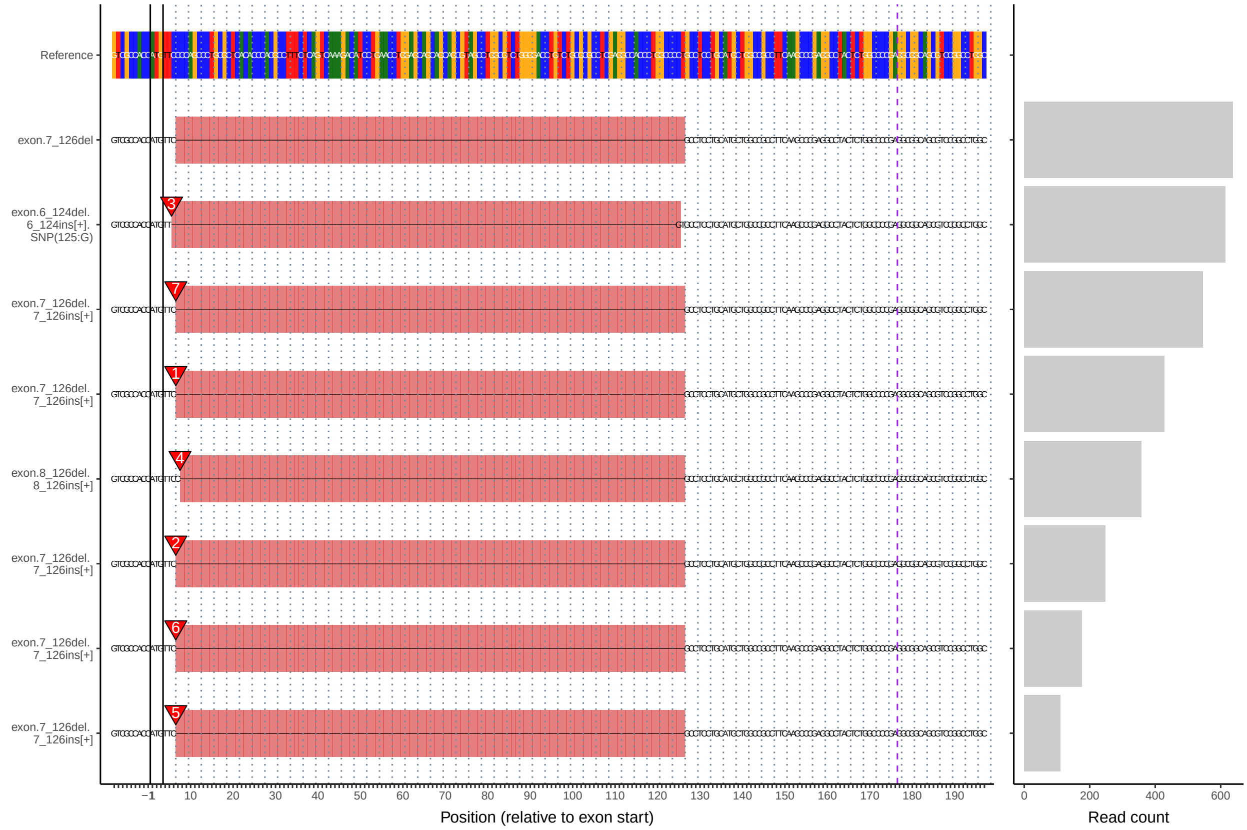

ID	Sequence
1	AAGCGCCATTCAGGATCACGTTACCCCGAAAAAAAGGTAC
2	80I
3	ACTGCGACG
4	59I
5	120I
6	100I
7	TTTAAACGTTGGTCCGTCAG

Fig E. Sequence alignment of a series of insertions (10 to 120) into a 120 bp deletion. If chimeric reads are excluded, only a few reads of the insertion of 20 bp in the gap were detected.

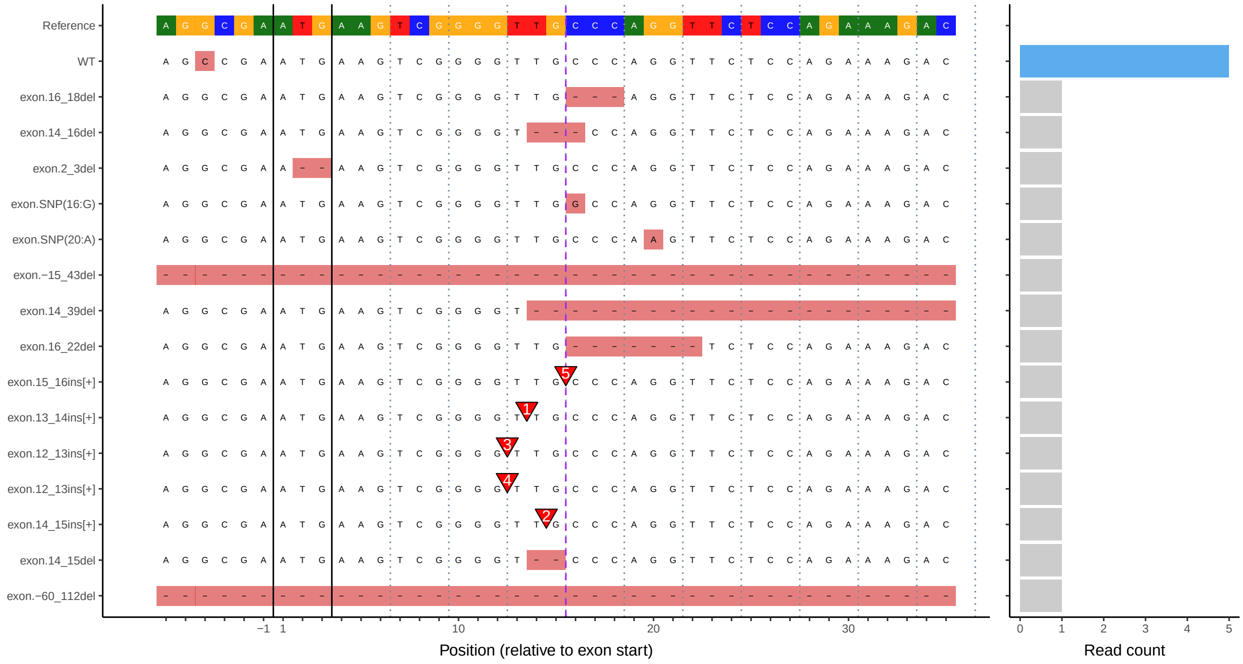

ID	Sequence
1	ATA
2	GTGGATTCCGGCATTTCTTA
3	TATATATATA
4	TATGCATCGA
5	TGC

Fig F. Sequence alignment of Synthetic data set 1 analysed by AlleleProfileR

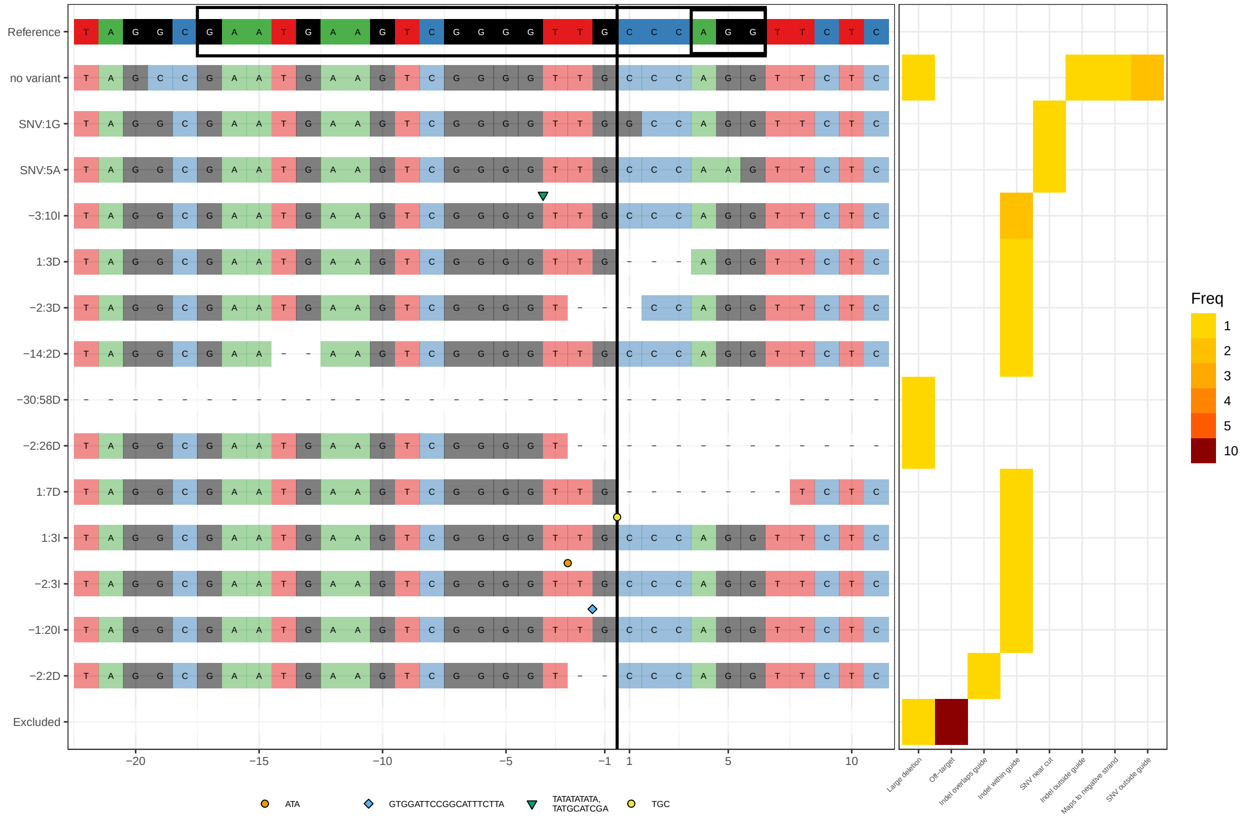

Fig G. Sequence alignment of Synthetic data set 1 analysed by CrispRVariants

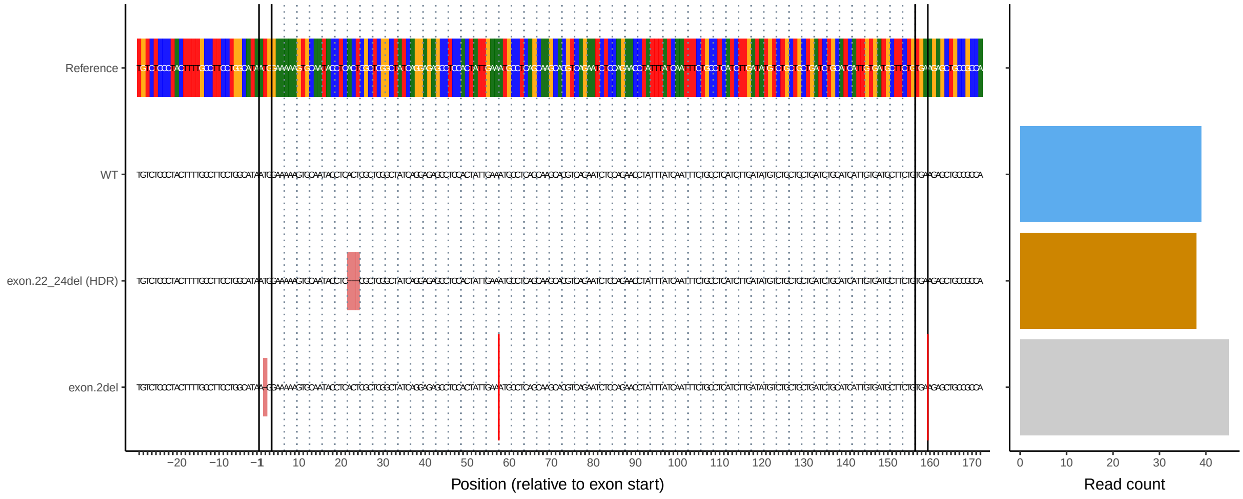

Fig H. Sequence alignment of a gene editing experiment with HDR template. The HDR allelic is colored orange.

```
## [[2]]
```

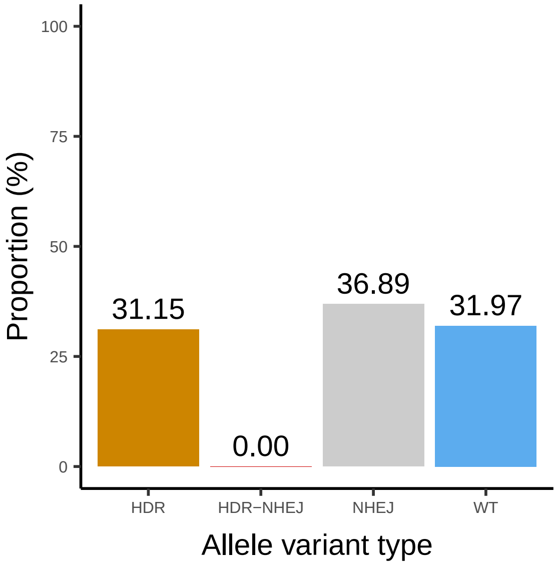

Fig I. Mutation types plot of Embryo4/PLN. Relative abundance of WT, NHEJ, HDR, and mixtures of HDR and NHEJ are quantified. This example has comparable levels of WT, NHEJ, and HDR, but no mix of HDR and NHEJ within the same read.

Premature stop codons and Cryptic variants

Indels or SNPs could generate total knock-out of a protein, mutated or truncated proteins. AlleleProfileR assesses the coding sequence for in frame stop codons. For example, Figure J represents the alignment of a 1 bp insertion in exon 2 of TBX5 resulting in an in frame stop codon (annotated by a red line) and hence a truncated protein.

```r
# plot alignments
AlleleProfileR.plot(crispr_config, 5, 3)
```

In addition to premature stop codons in frame with the start codon, indels may also result in the genesis of new cryptic coding sequences. AlleleProfileR identifies and reports such alternate coding sequences. For example, Figures K or L: nothwitstanding the disruption of the ATG codon, the genetic region contains a start codon in frame with the coding sequence. These alternate proteins may or may not be translated. The relevance of such cryptic proteins is difficult to predict, but such information is useful in designing experiments and interpreting biological results. The position of the cryptic start and stop positions are annotated by red lines. Multiple suitable pairs of start and stop codons may be present. In this case, AlleleProfileR will only report the cryptic allele with the most DNA sequence homology to the original sequence. Moreover, AlleleProfiler also considers the genetic sequence outside of the sequence range by importing the WT sequence.

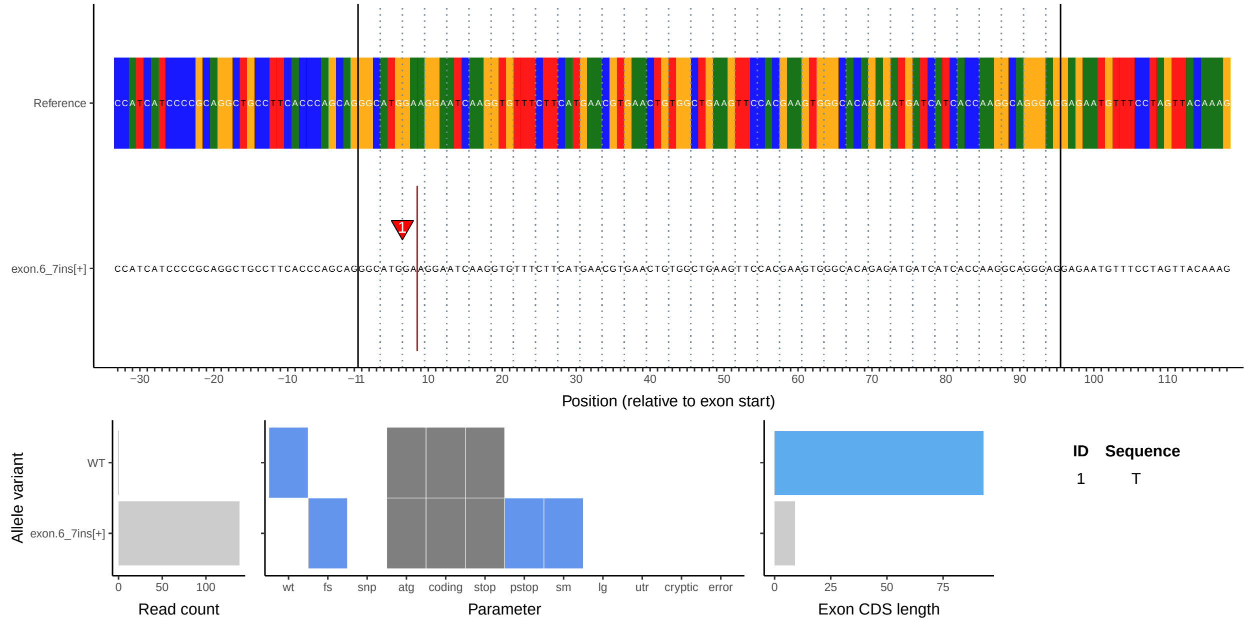

Fig J. Sequence alignment and characterization plot of mutant where an indel resulted in a frame shift and premature stop codon resulting in a truncated coding sequence.

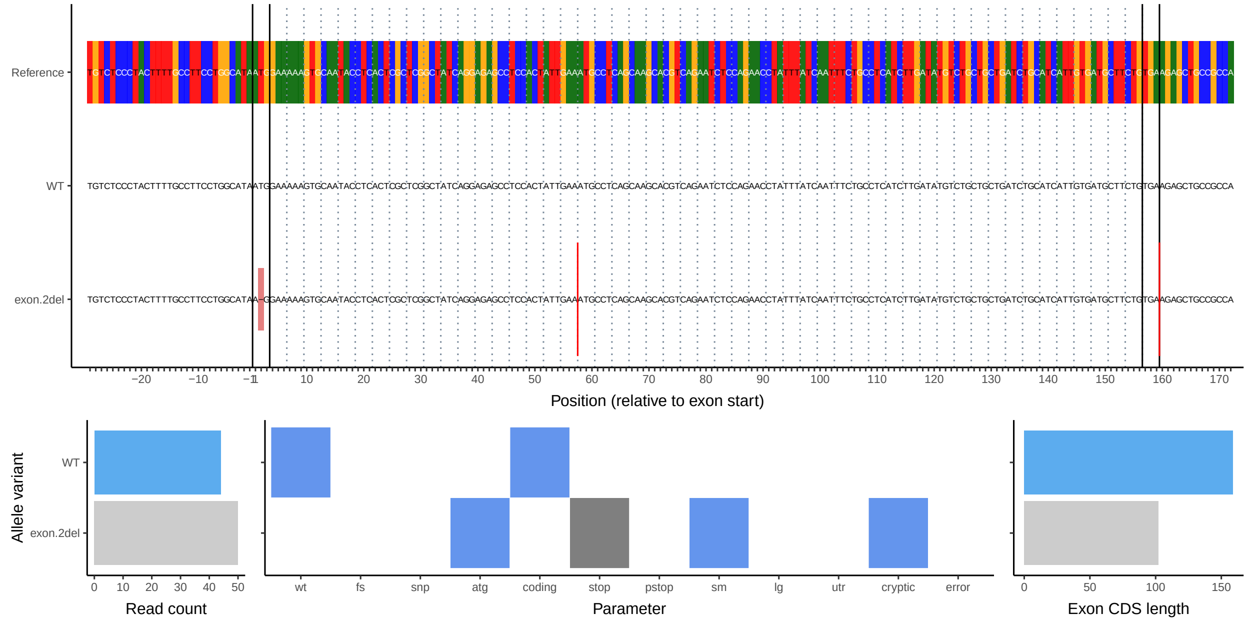

Fig K. Sequence alignment and characterization plot where the disruption of the start codon was rescued by an alternative start codon downstream resulting in a shorter cryptic coding sequence.

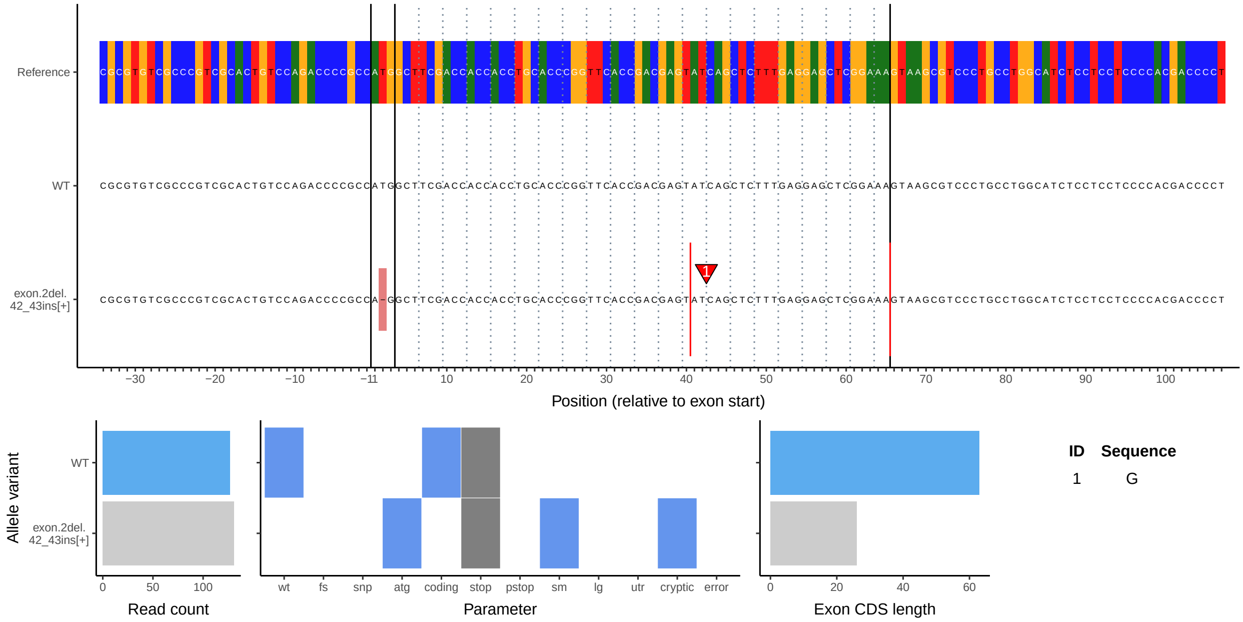

Fig L. Sequence alignment and characterization plot of mutant where the disruption of the start codon was rescued by an inserted start codon downstream in frame with the existing exon end, resulting in an alternate shorter cryptic exon.

Base editing

Base editing technologies, where a targeted DNA base is irreversibly converted into another without DNA cleavage or HDR template, have recently been reported. To illustrate AlleleProfileR's capabilities, we reanalyzed some of the data from the Gaudelli et al. study[9] describing adenine base editors (ABEs) that mediate the conversion of A • T to G • C in genomic DNA. Following code reproduces the display figure in the AlleleProfileR paper. Briefly, we will retrieve the NGS data for site 6 deposited by the authors in the NCBI Sequence Read Archive[10] database (Table D), preprocess the sequencing reads, reanalyze the reads using AlleleProfileR, generate summary statistics and plot the results (Figure M).

Step 1: Retrieve the published data from NCBI-SRA

```
# experiments table
SRAlist <- read.table("SRA_table_baseedit.txt", header=T, sep = "\t")

# retrieve data
for (exp in SRAlist$Experiment) {
  dir <- paste("files/input/", exp, sep="")
  cmd1 <- paste0("mkdir ", dir)
  message(cmd1, "\n");
  system(cmd1)
  cmd2 <- paste0("fastq-dump -A ", exp, " -O ", dir)
  message(cmd2, "\n");
  system(cmd2)
  ## masking filter
  cmd3 <- paste0("seqtk seq -q32 -n N ", dir, "/", exp, ".fastq > ",
```

[9]Gaudelli NM, Komor AC, Rees HA, Packer MS, Badran AH, Bryson DI, et al. Programmable base editing of A • T to G • C in genomic DNA without DNA cleavage. Nature. 551(7681), 464, 2017.
[10]https://www.ncbi.nlm.nih.gov/sra

Table D. Overview of site 6 data deposited in NCBI-SRA by Gaudelli et al.

AvgSpotLen	BioSample	Experiment	Library_Name	MBases	MBytes
225	SAMN07759268	SRX3260258	site 6_ABE7.3_Hek293T_rep 2	15	8
241	SAMN07759267	SRX3260259	site 6_ABE7.3_Hek293T_rep 1	25	15
186	SAMN07759266	SRX3260260	site 6_ABE7.2_Hek293T_rep 3	14	8
241	SAMN07759265	SRX3260261	site 6_ABE7.2_Hek293T_rep 2	2	1
243	SAMN07759264	SRX3260262	site 6_ABE7.2_Hek293T_rep 1	26	16
197	SAMN07759263	SRX3260263	site 6_ABE7.1_Hek293T_rep 3	15	8
241	SAMN07759262	SRX3260264	site 6_ABE7.1_Hek293T_rep 2	2	1
241	SAMN07759261	SRX3260265	site 6_ABE7.1_Hek293T_rep 1	26	15
238	SAMN07759270	SRX3260266	site 6_ABE7.4_Hek293T_rep 1	18	11
233	SAMN07759286	SRX3260217	site 6_ABE7.9_Hek293T_rep 2	18	9
241	SAMN07759285	SRX3260218	site 6_ABE7.9_Hek293T_rep 1	25	15
193	SAMN07759287	SRX3260220	site 6_ABE7.9_Hek293T_rep 3	16	9
243	SAMN07759282	SRX3260221	site 6_ABE7.8_Hek293T_rep 1	27	16
191	SAMN07759281	SRX3260222	site 6_ABE7.7_Hek293T_rep 3	15	9
195	SAMN07759284	SRX3260223	site 6_ABE7.8_Hek293T_rep 3	17	10
223	SAMN07759283	SRX3260224	site 6_ABE7.8_Hek293T_rep 2	13	7
239	SAMN07759279	SRX3260184	site 6_ABE7.7_Hek293T_rep 1	24	14
228	SAMN07759280	SRX3260185	site 6_ABE7.7_Hek293T_rep 2	17	8
227	SAMN07759277	SRX3260186	site 6_ABE7.6_Hek293T_rep 2	14	7
191	SAMN07759278	SRX3260187	site 6_ABE7.6_Hek293T_rep 3	15	8
187	SAMN07759275	SRX3260188	site 6_ABE7.5_Hek293T_rep 3	15	8
242	SAMN07759276	SRX3260189	site 6_ABE7.6_Hek293T_rep 1	24	14
240	SAMN07759273	SRX3260190	site 6_ABE7.5_Hek293T_rep 1	25	15
225	SAMN07759274	SRX3260191	site 6_ABE7.5_Hek293T_rep 2	16	8
224	SAMN07759271	SRX3260192	site 6_ABE7.4_Hek293T_rep 2	12	6
195	SAMN07759272	SRX3260193	site 6_ABE7.4_Hek293T_rep 3	15	8
203	SAMN07759290	SRX3260215	site 6_ABE7.10_Hek293T_rep 3	29	15
189	SAMN07759289	SRX3260216	site 6_ABE7.10_Hek293T_rep 2	4	2
214	SAMN07759288	SRX3260219	site 6_ABE7.10_Hek293T_rep 1	8	4

Table E. Configuration table of the genes of interest

Gene	Chr	Start	Stop	ATG	StopCodon	StartType	StartShift	StopType	StopShift	PCRStart	PCRStop	CutSites
bpedit	chr9	300	350	315	338	I	0	I	0	10	990	325

```
                 dir, "/", exp, ".assembled.fastq")
  message(cmd3, "\n");
  system(cmd3)
}
```

Step 2: Configuration

AlleleProfileR was provide following configuration of the gene of interest (Table E), and a reference a small region surrounding the on-target region of site 6 was used:

```
> chr9
GTCTCAAAACTAATCTTGTAGCTACGCCTGTGATGGGCTAATTGATGAATCAGTGCTGGAGAATGGGTCACAGTGGCAAATGAGGCTG
GAGAGGCCCGTGAGCTGCTGTCCCAGAGGGCCTGGGATGTCCTGCAGGGAGCTTGGCATGAGAAACCTTGGAGAGTTTTAAGCAAGGG
CTGATGTGGGCTGCCTAGAAAGGCATGGATGAGAGAAGCCTGGAGACAGGGATCCCAGGGAAACGCCCATGCAATTAGTCTATTTCTG
CTGCAAGTAAGCATGCATTTGTAGGCTTGATGCTTTTTTTCTGCTTCTCCAGCCCTGGCCTGGGTCAATCCTTGGGGCCCAGACTGAG
CACGTGATGGCAGAGGAAAGGAAGCCCTGCTTCCTCCAGAGGGCGTCGCAGGACAGCTTTTCCTAGACAGGGGCTAGTATGTGCAGCT
CCTGCACCGGGATACTGGTTGACAAGTTTGGCTGGGCTGGAAGCCAGCACCTAGGGAGGTCCCTGGAAGGGGCCAGCCTCACCAGGAG
AGGAGGGACCTGGCCCTTCAGGGTCGAGCTCAACAGAGGAAAAGATCTCAGGGCACCCAGAGCCCAGTGGCTTTCAGCACCTGCATGA
AAATCAGAGATCAACCAGATTACCCCATATTGCCCAAGAGAAAACTGAGGCCAGAAAGTGATGGAGCTTGCCCAGGACCCCAGAGGGT
GTTAGAGGCAGGCCAGAACCAGAGGACAGCTCTGTCCCAGCACTTGCCCTCCTTTAATTAGGTGGGTGGTCATATCGCAGTGGTCAGA
GACATTGCAAACAATAATAATAAATATTGAGCACTTGTTACACGCAGGGCACTATGCTAAGCACCTTGTATTTGTTATGTCCTTTCAT
CCTAGCAACTTCTCTGGAAGTGGAGTAATATCTCCCCCATGTCCTACATAAGACAATGGATACACTGGAGGGTTAAAGCCATGCCTTG
CCTAAAGTTACACAGCTGTGCTTAACAGCTGGA
```

Step 3: Preprocess the reads

```
# read folders and prepreocess
besamples <- AlleleProfileR.read.folders(type = "fastq")
AlleleProfileR.preprocess(besamples, index = "files/index/index_baseedit.fa",
                          method.qc = "none", params.qc = "",
                          method.merge = "none", params.merge = "",
                          method.map = "bwa", subset = c(10:38))
```

Configuration:

```
# set configuration
besamples <- AlleleProfileR.read.folders(type = "bam")
becrispr_config <- AlleleProfileR.setup(samples = besamples,
                                        genes = "files/config/be_genes.csv",
                                        index = "files/index/index_baseedit.fa",
                                        cutoff = 0,
                                        ignore.snp = F,
                                        cut.range = 0,
                                        ignore.single = T,
                                        cutoff.large = 25,
                                        chimeric.max.insertion.size = 50,
                                        suppress.messages = F)
```

Step 4 : Execute analysis

```
# process alleles
AlleleProfileR.batch(becrispr_config, cores=3, subset=list(c(10:38),c(1)), summarize=TRUE)
```

Step 5: Generate summary statistics

```
# experiments table
SRAlist <- read.table("SRA_table_baseedit.txt", header=T, sep = "\t")
# extract the interesting data
beoutput <- data.frame(matrix(nrow = 0, ncol = 5))
colnames(beoutput) <- c("Exp", "Treatment", "Group", "Value", "Measurement")
outputoutline <- data.frame(Exp=paste(SRAlist$Experiment), Treatment=paste(SRAlist$treatment))

poss <- c(329,333,325)
meas <- c("On-target","Off-target-1","Off-target-2")

for (i in 1:dim(outputoutline)[1]) {
  tempgroup <- paste(gsubfn::strapplyc(paste(outputoutline$Treatment[i]),
                                      "_ABE([[:digit:]]+).([[:digit:]]+)_")[[1]],
                     collapse=".")
  tempsample <- which(besamples$Sample == paste(outputoutline$Exp[i]))
  tempresultsmain <- AlleleProfileR.plot.seqlogo(becrispr_config,
                                                  sample.id = tempsample, gene.id = 1,
                                                  plot = F, customrange = c(316,336))

  # prep output
  tempoutput <- data.frame(Exp=paste(outputoutline$Exp[i]),
                           Treatment=paste(outputoutline$Treatment[i]),
                           Group=tempgroup, Value=c(NA,NA,NA),
                           Measurement=c(NA,NA,NA))
  for (j in 1:dim(tempoutput)[1]) {
    tempresults <- tempresultsmain[which(tempresultsmain$abspos == poss[j]),]
    tempoutput$Value[j] <- 100*tempresults[,"C"]/sum(tempresults[,c(4:7)])
    tempoutput$Measurement[j] <- meas[j]
  }

  beoutput <- rbind(beoutput,tempoutput)
}

# summary
library(Rmisc)
summary <- summarySE(beoutput, measurevar="Value",
                     groupvars=c("Group","Measurement"))
summary$Measurement <- factor(summary$Measurement,
                              levels = c("On-target", "Off-target-1", "Off-target-2"))
summary$Group <- factor(summary$Group,
                        levels = c("7.1", "7.2", "7.3", "7.4",
                                   "7.5", "7.6", "7.7", "7.8",
                                   "7.9", "7.10"))
```

Step 6: Plot

We used ggplot2 to generate a barchart of the on- and off-target base editing efficacy for site 6 for the various ABEs deposited on SRA (Figure M). Additionally, the sequence logo plot provides a convenient graphical representation of both on-target as well as off-target base editing events (Figure N). The results match well with those obtained using the Gaudelli script (Figure O).

```
# bar chart
ggplot(summary, aes(x=Group, y=Value, fill=Measurement)) +
  geom_bar(position=position_dodge(), stat="identity")+
  geom_errorbar(aes(ymin=Value-se, ymax=Value+se), width=.2, position=position_dodge(.9)) +
  theme_classic() + xlab("ABE") + scale_y_continuous(name ="% seq reads (A/T to G/C converted)",
  breaks=c(0,5,10,20,30,40,50,60,70)) + theme(legend.position="none")
```

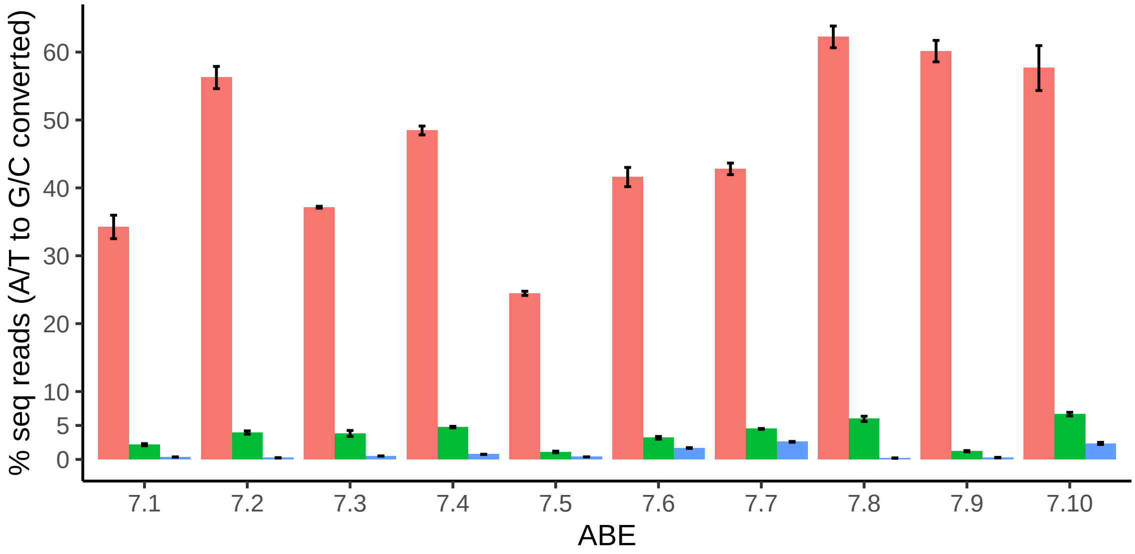

Fig M. On- and off-target base editing efficacy for site 6 for the various ABEs deposited on SRA. On-target base editing (pink), off-target edits at two sites (green: off-target 1, blue: off-target 2). The bar charts represent mean +/- standard error (three replicates).

```
# sequence logo plot
AlleleProfileR.plot.seqlogo(becrispr_config, 20, 1, plot = T, customrange = c(324,334))
```

```
## NULL
```

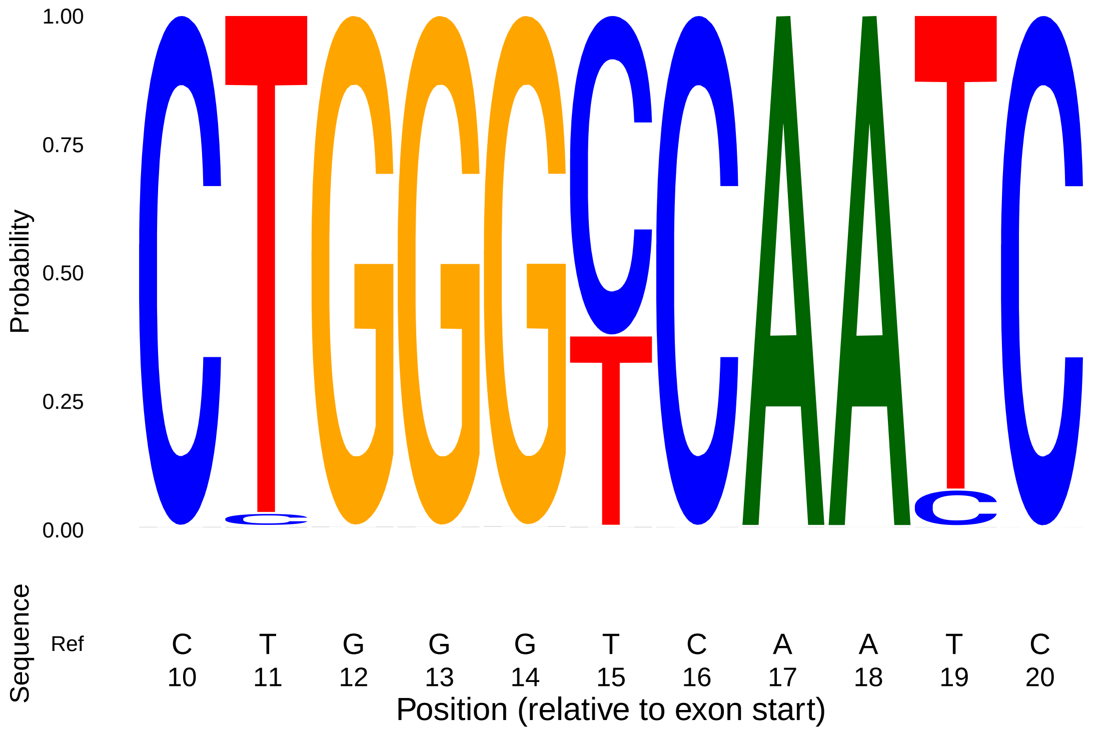

Fig N. Sequence logo plot of site 6 ABE7.10 Hek293T rep 3 (SRX3260215)

Appendix

Session information

```
sessionInfo()
```

```
## R version 3.6.1 (2019-07-05)
## Platform: x86_64-apple-darwin15.6.0 (64-bit)
## Running under: macOS Catalina 10.15.2
##
## Matrix products: default
## BLAS:   /Library/Frameworks/R.framework/Versions/3.6/Resources/lib/libRblas.0.dylib
## LAPACK: /Library/Frameworks/R.framework/Versions/3.6/Resources/lib/libRlapack.dylib
##
## locale:
## [1] en_US.UTF-8/en_US.UTF-8/en_US.UTF-8/C/en_US.UTF-8/en_US.UTF-8
##
## attached base packages:
## [1] stats4    parallel  stats     graphics  grDevices utils     datasets
## [8] methods   base
##
## other attached packages:
##  [1] Rmisc_1.5                          plyr_1.8.5
##  [3] lattice_0.20-38                    GenomicAlignments_1.20.1
##  [5] Rsamtools_2.0.3                    SummarizedExperiment_1.14.1
##  [7] DelayedArray_0.10.0                BiocParallel_1.18.1
##  [9] matrixStats_0.55.0                 Biobase_2.44.0
```

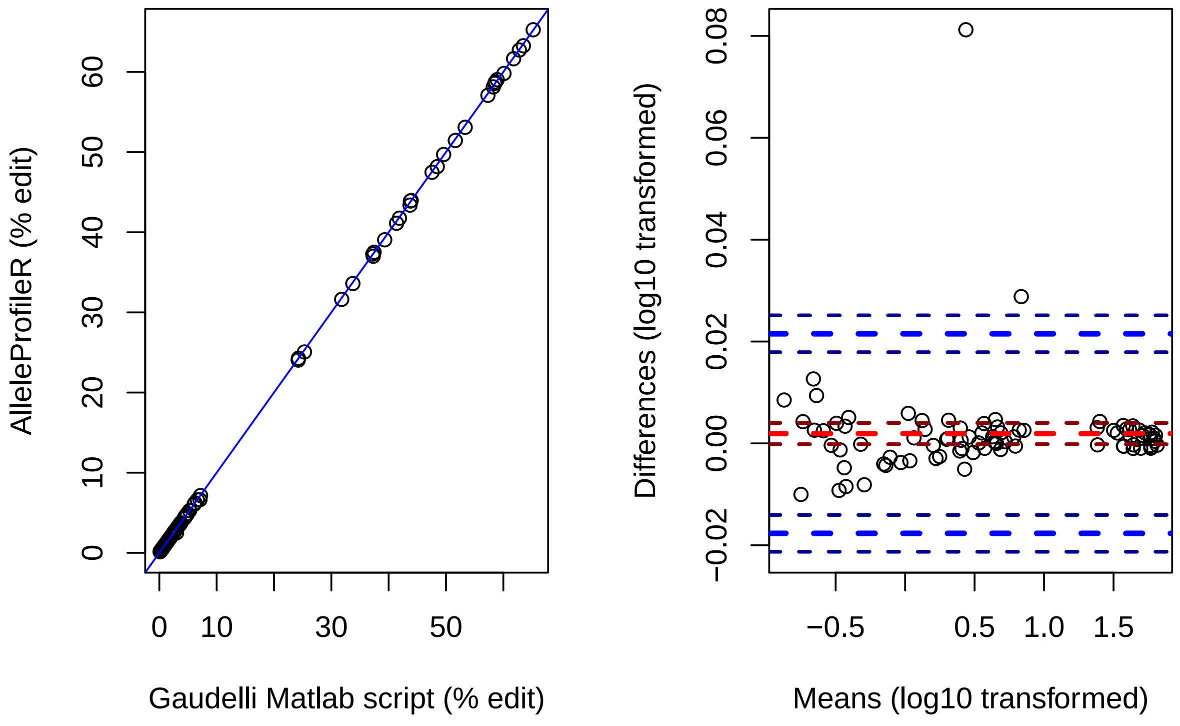

Fig O. Comparison of the output of the Gaudelli base calling script with AlleleProfiler. (l) Correlation between the results of the Gaudelli Matlab script and AlleleProfileR. (r) Bland Altman Plot comparing the two methods with respect to the results acquired using the Gaudelli Matlab script.

```
## [11] CrispRVariants_1.12.0             BSgenome.Drerio.UCSC.danRer7_1.4.0
## [13] BSgenome_1.52.0                   rtracklayer_1.44.4
## [15] Biostrings_2.52.0                 XVector_0.24.0
## [17] GenomicRanges_1.36.1              GenomeInfoDb_1.20.0
## [19] IRanges_2.18.3                    S4Vectors_0.22.1
## [21] BiocGenerics_0.30.0               knitr_1.26
## [23] ggplot2_3.2.1                     AlleleProfileR_0.1.1.0000
## [25] kableExtra_1.1.0                  magrittr_1.5
##
## loaded via a namespace (and not attached):
##  [1] httr_1.4.1             bit64_0.9-7            viridisLite_0.3.0
##  [4] gsubfn_0.7             assertthat_0.2.1       blob_1.2.0
##  [7] GenomeInfoDbData_1.2.1 yaml_2.2.0             BlandAltmanLeh_0.3.1
## [10] RSQLite_2.1.4          pillar_1.4.2           backports_1.1.5
## [13] glue_1.3.1             digest_0.6.23          ggsignif_0.6.0
## [16] rvest_0.3.5            colorspace_1.4-1       cowplot_1.0.0
## [19] htmltools_0.4.0        Matrix_1.2-18          XML_3.98-1.20
## [22] pkgconfig_2.0.3        ggseqlogo_0.1          zlibbioc_1.30.0
## [25] purrr_0.3.3            scales_1.1.0           webshot_0.5.2
## [28] tibble_2.1.3           farver_2.0.1           ggpubr_0.2.4
## [31] withr_2.1.2            lazyeval_0.2.2         proto_1.0.0
## [34] crayon_1.3.4           memoise_1.1.0          evaluate_0.14
## [37] xml2_1.2.2             tools_3.6.1            hms_0.5.2
## [40] lifecycle_0.1.0        stringr_1.4.0          munsell_0.5.0
## [43] AnnotationDbi_1.46.1   compiler_3.6.1         rlang_0.4.2
## [46] grid_3.6.1             RCurl_1.95-4.12        rstudioapi_0.10
## [49] bitops_1.0-6           tcltk_3.6.1            labeling_0.3
## [52] rmarkdown_2.0          gtable_0.3.0           DBI_1.0.0
## [55] reshape2_1.4.3         R6_2.4.1               gridExtra_2.3
## [58] dplyr_0.8.3            bit_1.1-14             zeallot_0.1.0
## [61] readr_1.3.1            stringi_1.4.3          Rcpp_1.0.3
## [64] vctrs_0.2.0            tidyselect_0.2.5       xfun_0.11
```
